# Centuries of genome instability and evolution in soft-shell clam, *Mya* *arenaria*, bivalve transmissible neoplasia

**DOI:** 10.1038/s43018-023-00643-7

**Published:** 2023-10-02

**Authors:** Samuel F. M. Hart, Marisa A. Yonemitsu, Rachael M. Giersch, Fiona E. S. Garrett, Brian F. Beal, Gloria Arriagada, Brian W. Davis, Elaine A. Ostrander, Stephen P. Goff, Michael J. Metzger

**Affiliations:** 1 Pacific Northwest Research Institute, Seattle, WA USA; 2 Molecular and Cellular Biology Program, University of Washington, Seattle, WA USA; 3 Division of Environmental and Biological Sciences, University of Maine at Machias, Machias, ME USA; 4 Downeast Institute, Beals, ME USA; 5 Instituto de Ciencias Biomedicas, Facultad de Medicina y Facultad de Ciencias de la Vida, Universidad Andres Bello, Santiago, Chile; 6 FONDAP Center for Genome Regulation, Santiago, Chile; 7 Department of Veterinary Integrative Biosciences, Texas A&M University School of Veterinary Medicine, College Station, TX USA; 8 Department of Small Animal Clinical Sciences, Texas A&M University School of Veterinary Medicine, College Station, TX USA; 9 Cancer Genetics and Comparative Genomics Branch, National Human Genome Research Institute, National Institutes of Health, Bethesda, MD USA; 10 Department of Biochemistry and Molecular Biophysics, Columbia University, New York, NY USA; 11 Department of Microbiology and Immunology, Columbia University, New York, NY USA

**Keywords:** Cancer, Genetics, Evolution

## Abstract

Transmissible cancers are infectious parasitic clones that metastasize to new hosts, living past the death of the founder animal in which the cancer initiated. We investigated the evolutionary history of a cancer lineage that has spread though the soft-shell clam (*Mya* *arenaria*) population by assembling a chromosome-scale soft-shell clam reference genome and characterizing somatic mutations in transmissible cancer. We observe high mutation density, widespread copy-number gain, structural rearrangement, loss of heterozygosity, variable telomere lengths, mitochondrial genome expansion and transposable element activity, all indicative of an unstable cancer genome. We also discover a previously unreported mutational signature associated with overexpression of an error-prone polymerase and use this to estimate the lineage to be >200 years old. Our study reveals the ability for an invertebrate cancer lineage to survive for centuries while its genome continues to structurally mutate, likely contributing to the evolution of this lineage as a parasitic cancer.

## Abstract (web summary)

Hart et al. study the transmissible marine leukemia of the soft-shell clam *Mya* *arenaria* and focus on a cancer lineage that is characterized by varied genomic instability as well as a mutational signature that includes an error-prone polymerase.

## Main

Most cancers arise from oncogenic mutations in host cells and remain confined to the body of that host; however, a small number of transmissible cancer lineages exist in which cancer cells metastasize repeatedly to new hosts, living past the death of their original hosts as asexually reproducing unicellular organisms^1^. Observed cases of transmissible cancer in nature include canine transmissible venereal tumor (CTVT) in dogs^2,3^, two unrelated lineages of devil facial tumor disease (DFTD) in Tasmanian devils^4,5^ and at least eight bivalve transmissible neoplasia (BTN) lineages observed in several marine bivalve species^6–10^. Although transmissible cancers and their host genomes have been well characterized in dogs^11–13^ and Tasmanian devils^14–16^, little is known about the evolutionary history of the BTN lineages, which have only recently been recognized as transmissible cancers. Here we perform a genome-wide analysis of a BTN lineage found in the soft-shell clam (*Mya* *arenaria*) or MarBTN.

**Fig. 1:**
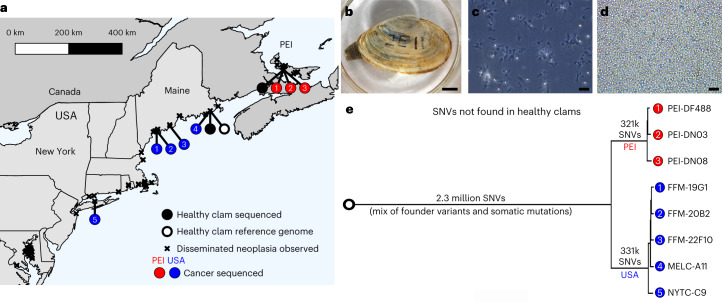
MarBTN distribution and sequencing. **a**, Locations of samples sequenced (circles) and disseminated neoplasia observations (indicated by x) along the east coast of North America. Circles colored for healthy clams (black) and MarBTN sampled from the PEI (red) or USA (blue) coast. **b**,**c**, Image of healthy clam used to assemble reference genome (MELC-2E11) (**b**) and hemolymph of the same clam (**c**), with hemocytes extending pseudopodia. The healthy reference clam (open black circle from **a**) was included in WGS analysis. **d**, Hemolymph from a clam infected with MarBTN (FFM-22F10), with distinct rounded morphology and lack of pseudopodia of cancer cells (representative of similar images from *n* = 8 MarBTN samples in this study). Scale bars, 10 mm (clam) and 50 µm (hemolymph). **e**, Phylogeny of cancer samples built from pairwise differences of SNVs not found in healthy clams, excluding regions that show evidence of LOH. Numbers along branches indicate the number of SNVs unique to and shared by individuals in that clade. All nodes have 100 of 100 bootstrap support. Source data

BTN is a fatal leukemia-like cancer characterized by high numbers of cancer cells in the circulatory fluid of the bivalve and dissemination into tissues in the later stages of disease. BTN cells can survive for days to weeks in seawater^17,18^ and likely spread from animal to animal by transmission through the water column. This cancer, referred to in the literature as disseminated neoplasia or hemic neoplasia, was first reported in soft-shell clams in the 1970s^19,20^ and has since been found across much of the soft-shell clam’s native range along the east coast of North America (Fig. 1a). In the 1980s in New England and in the 2000s in Prince Edward Island, Canada, severe outbreaks were documented with prevalence as high as 90% followed by severe population losses^21,22^. The disease is still observed throughout this range, although no more recent large-scale population die-offs have been reported. All disseminated neoplasia isolates tested in a 2015 study were shown to be of clonal origin and it was hypothesized that historical observations of the cancer dating back to the 1970s were occurrences of this same clonal lineage^6^; however, it is not known how long this lineage has propagated, or how the genome has evolved since the original cancer initiated. To address these and other questions, we assembled a high-quality soft-shell clam reference genome and characterized the genome evolution of the MarBTN lineage by comparative analysis of healthy clam and MarBTN sequences. We show a notable pattern of mutation occurrence and evolution, suggestive of an unstable genome with the potential to rapidly mutate despite its long-term survival.

## Results

### Sample sequencing and genome assembly

We assembled a soft-shell clam reference genome from a single healthy female clam collected from Larrabee Cove, Machiasport, Maine, USA (Fig. 1b,c; MELC-2E11). We assembled PacBio long reads into contigs using FALCON-Unzip^23^, scaffolded contigs to the chromosome-level with Hi-C sequences using FALCON-Phase, polished the scaffolds using 10x Chromium reads and annotated with RNA-seq reads using MAKER to yield a high-quality reference genome. The final reference genome is 1.22 Gb, organized into 17 phased scaffolds, matching the 17 chromosomes expected based on karyotype data^24^. The contig N50 is 3.4 Mb and the metazoan BUSCO (Benchmarking Universal Single Copy Orthologs^25^) score is 94.9%. Our assembly is similar in size, GC and repeat content of a recently published *M.* *arenaria* genome^26^ but with drastically improved contiguity and completeness (Supplementary Table 1), allowing for comprehensive genomic investigation into the evolutionary history of MarBTN.

**Extended Data Fig. 1:**
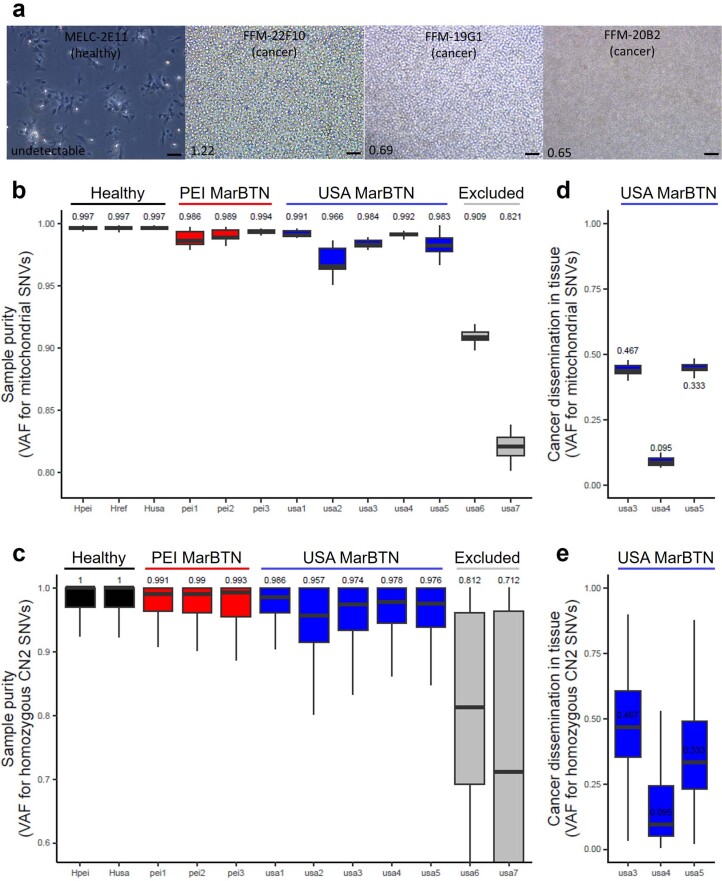
Minimal host DNA is found in cancer hemolymph samples. **(a)** Hemolymph images for the four clams in this study sampled 2018–21. The other seven clams sampled 2010–14 were reported in past studies by Arriagada & Metzger et al. (2014) and Metzger et al (2015). Scale bars are 50 µm. Fraction of cancer cells detected by MarBTN-specific qPCR, as reported by Giersch et al. (2022), are included in the lower left of each image. Note that while this assay is highly sensitive for the detection of low levels of MarBTN infection in animals, the fraction is a ratio of two qPCR values and minor variation in qPCR values can lead to large variation in the fraction when it is close to 100% cancer. **(b)** We identified SNVs in mitochondrial DNA in each individual sample and used the median VAF of those SNVs to estimate the purity of the sample. Number of loci: 21, 20 and 13 for healthy clams as ordered in figure, 53 (PEI) and 46 (USA) likely somatic for MarBTN samples. **(c)** Since mitochondrial genome copy numbers may differ between host and MarBTN cells, we also identified homozygous nuclear SNVs in regions called as copy number 2 in both sub-lineages and used the median VAF of those SNVs to estimate the purity of the sample (number of loci: 250,000 for non-reference healthy clams, 15,000 MarBTN-specific loci for MarBTN samples). Values for pure samples would be expected to be slightly below one due to mapping/sequencing errors, as evidenced by the healthy clams, which serve as pure sample controls (black, all DNA is from one individual). In cancer samples, deviation below this near-one value is attributed to the presence of contaminating host DNA (DNA is a mixture of two individuals – the cancer and the host). Two MarBTN isolates that were excluded from this study due to high host DNA contamination are included on this plot as contaminated sample controls (gray). Both nuclear and mitochondrial markers calculations yield similar estimates of cancer cell purity 96% or greater. MtDNA has the advantage of all loci being ‘homozygous’ and much greater depth than nuclear, giving more resolution as to the exact cancer cell percentage. However, mtDNA copies per cell may vary from sample to sample and between host and cancer. We also extracted DNA from tissue samples for a subset of the USA cancers and estimated the fraction of cancer DNA disseminated into tissue using the same methodology for mitochondrial **(d)** and nuclear **(e)** loci. Tissue samples contain variable and in some cases quite high, fractions of cancer DNA. This made genome-wide differentiation between host and cancer SNVs difficult in tissue and lead us to not include paired tissue DNA in our analyses, instead relying on variant calling thresholds to eliminate host variants from our cancer variant calling pipelines. Box plots display ggplot defaults - median (center), interquartile range (box), and the less extreme of minima/maxima or 1.5* interquartile range (whiskers). Source data

We performed whole-genome sequencing (WGS) on three healthy uninfected clams and eight isolates of MarBTN from the hemolymph of highly infected clams (for example Fig. 1d) sampled from five locations across the established MarBTN range^27^ (Fig. 1a and Supplementary Table 2) and called single-nucleotide variants (SNVs) against the reference genome. Contaminating host variants were removed from MarBTN sequences via variant calling thresholds, rather than using paired tissue sequences as has been conducted for other transmissible cancers, as MarBTN hemolymph isolates were of high purity (>96% cancer DNA), whereas paired tissue samples from the host often contained high cancer DNA due to dissemination (Extended Data Fig. 1).

To investigate somatic evolution of the MarBTN lineage, it is important to distinguish between founder variants, those present in the genome of the founder clam from which the cancer initially arose, and somatic mutations, which occurred during the propagation and evolution of the cancer lineage. We observed that 10.7 million SNVs were shared by all MarBTN samples but not present in the reference genome. Of these, 8.1 million were found in at least one of the three healthy clams, indicating that these variants are likely from the germline of the founder.

**Extended Data Fig. 2:**
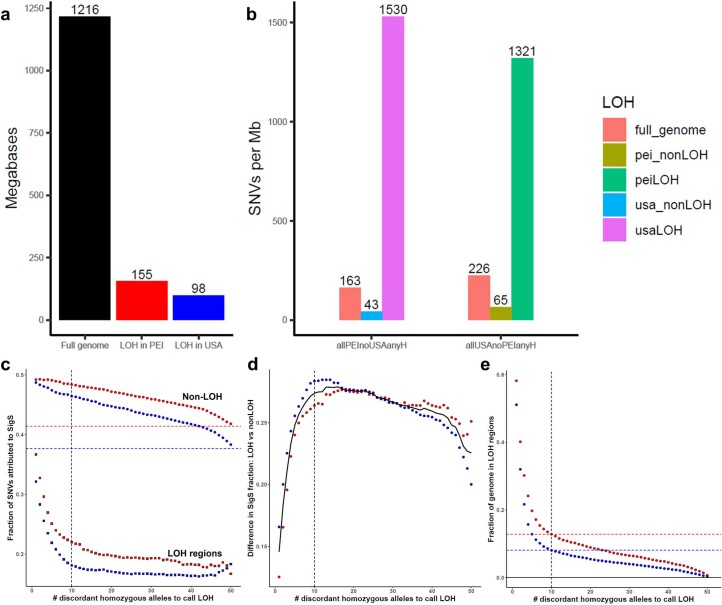
Loss of heterozygosity regions have sub-lineage-specific founder variants. **(a)** Comparative sizes of the assembled genome and the fractions called as LOH in the PEI (red) and USA (blue) sub-lineages. **(b)** SNV density of sub-lineage-specific founder variants (variants found in a healthy clam and all individuals of one sub-lineage but none in the other sub-lineage) across the genome and LOH regions called in the other sub-lineage. Density is 36× greater for PEI mutations in USA LOH regions versus non-LOH regions and 20x greater for USA mutations in PEI LOH regions versus non-LOH regions. LOH regions were ignored for somatic mutation analysis to reduce the influence of remaining founder variants in sub-lineage specific SNVs, which should otherwise consist of somatic mutations. **(c)** We used various thresholds of stringency to call LOH across the genomes of each sub-lineage based on the number of shared SNVs that were homozygous in one sub-lineage but heterozygous in the other across a window of 50 SNVs (x-axis). After calling LOH, we calculated the fraction of likely somatic mutations attributed to signature S in LOH (squares) and non-LOH (circles) (y-axis). Values are shown separately for the BTN subgroups from USA (blue) and PEI (red). Vertical dashed line indicates the threshold used for LOH-calling. Horizontal dashed lines indicated baseline signature S fractions without LOH region removal. **(d)** Plot of the difference between non-LOH and LOH regions as shown in (c) (calculated by subtracting the square from the circle). Black line shows the average difference, which peaks around the threshold used (10). **(e)** Proportion of the genome that is called LOH for each sub-lineage based on calling threshold. Dashed lines indicate the fraction of the genome called as LOH for each sub-lineage for the final threshold used. Source data

A MarBTN phylogeny, built from pairwise SNV differences between samples, confirmed the previous analysis identifying two distinct sub-lineages of MarBTN^6^, here referred to as the Prince Edward Island (PEI) and United States of America (USA) sub-lineages (Fig. 1e). While the original founder clam is lost, we are able to leverage this deep split between the sub-lineages to identify those mutations likely to be somatic and not founder, as SNVs that occurred after the divergence of the two subgroups would be somatic. Most SNVs identified in the cancers and also found in healthy animals (and therefore highly likely to be founder variants) were present in both sub-lineages of MarBTN, but we observed some genomic regions with clusters of these founder SNVs in one sub-lineage but not the other. These are unlikely to be somatic mutations, instead they likely indicate loss-of-heterozygosity (LOH) events that took place after divergence of the sub-lineages. LOH was identified in 8% and 13% of the USA and PEI sub-lineage genomes, respectively (Extended Data Fig. 2). LOH regions were excluded during identification of somatic mutations in the following SNV analysis unless otherwise noted, since we are unable to determine which mutations are founders and which are somatic in these regions. SNVs found in all cancer samples, but no healthy samples, represent a mix of both founder variants and somatic mutations (2.3 million), whereas SNVs found in just one or the other sub-lineage represent likely somatic mutations (700,242). The majority of these SNVs were shared by all individuals in a sub-lineage and are herein referred to as ‘high-confidence somatic mutations’ (320,715 for PEI and 331,167 for USA).

### Mutational biases in MarBTN

**Fig. 2:**
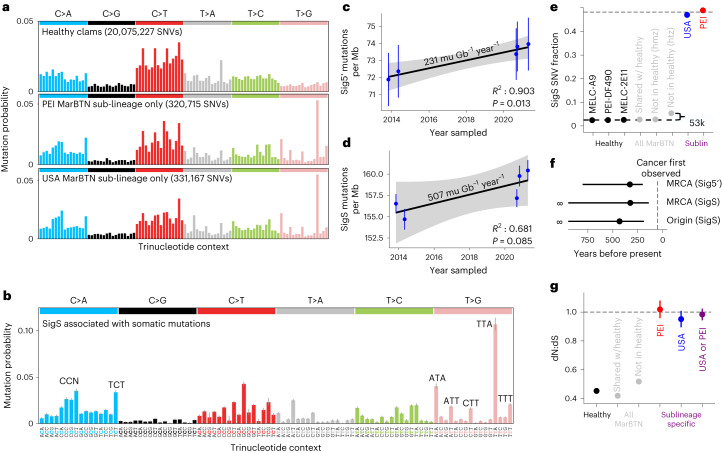
Unique mutational signature found in somatic mutations dates cancer to >200 years old. **a**, Trinucleotide context of SNVs found in healthy clams (top) and high-confidence somatic mutations in PEI (middle) or USA (bottom) sub-lineages, corrected for mutational opportunities in the clam genome. The trinucleotide order is the same as in **b**. **b**, De novo extracted mutational biases for SigS. **c**,**d**, Sig5′ (**c**) and SigS (**d**) attributed mutations per Mb (signature fitting estimates with fitting error) across USA MarBTN samples (*n* = 5) by sampling date. Results of linear regression with 95% CI (gray) overlaid. SNVs found in healthy clams, PEI MarBTN samples or LOH regions are excluded. **e**, Fraction of SNVs attributed to SigS from healthy clams (black), variants found in all MarBTN samples (gray) and high-confidence somatic mutations (colored). Variants found in all MarBTN samples are divided by whether they are found in healthy clams and whether they are homozygous (hmz) or heterozygous (htz). Dashed lines display SigS fraction estimates for likely somatic mutations and likely founder variants. **f**, Age estimate of the most recent common ancestor (MRCA) of the USA and PEI sub-lineages using Sig5′ and SigS and of the BTN origin from SigS mutations. **g**, dN:dS ratios (ratio of 1 indicates neutrality) for SNVs found in healthy clams (black), SNVs found in all MarBTN samples (gray) and high-confidence somatic mutations (colored) (*n* = 20,075,227, 7,676,209, 2,596,657, 320,715, 331,167 and 651,882 as shown from left to right). Error bars in all plots display 95% CI. Source data

**Extended Data Fig. 3:**
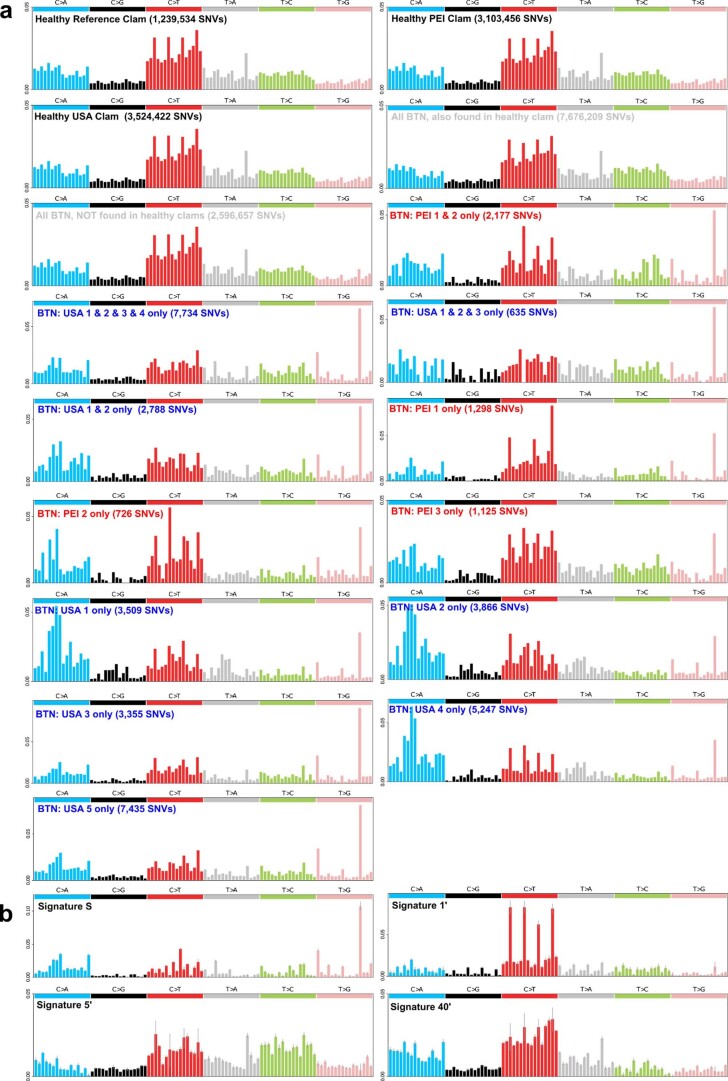
Raw mutational spectra and *de* novo extracted mutational signatures. **(a**) Plots show the mutational probability of SNVs in all trinucleotide contexts that were identified in various samples after filtering. Trinucleotide order is the same as shown in Fig. 2. Healthy clam SNVs (black labels - top) refer to SNVs that were unique to that clam and not found in other clams, resulting in no overlap of SNVs but still very similar spectra. SNVs found in all BTN samples (gray labels – upper middle) are divided into those found in a healthy clam (likely all from the founder clam genome) and those not found in any of the three healthy clams (includes a mixture of founder and early somatic mutations). Likely somatic SNVs found within the USA (blue labels) and PEI (red labels) sub-lineages show those SNVs that are either shared between all samples (Fig. 2a - not shown here), multiple samples (lower middle), or unique to individual samples (bottom). SNVs found in All mutational probabilities are corrected for mutational opportunities in the clam genome, and total mutation counts in each image are shown in the label. **(b)** We performed de novo mutational signature extraction to identify trinucleotide SNV differences between the various samples in this study, yielding four mutational signatures with mutational probabilities corrected for mutational opportunities in the clam genome. Error bars display 95% confidence intervals as determined by the extraction software, sigfit. Signatures sig1’, sig5’ and sig40’ are named after the closest signature in the COSMIC database, as determined by cosine similarity. SigS was named to reflect that it was specific to Somatic mutations in cancer samples. Source data

**Extended Data Fig. 4:**
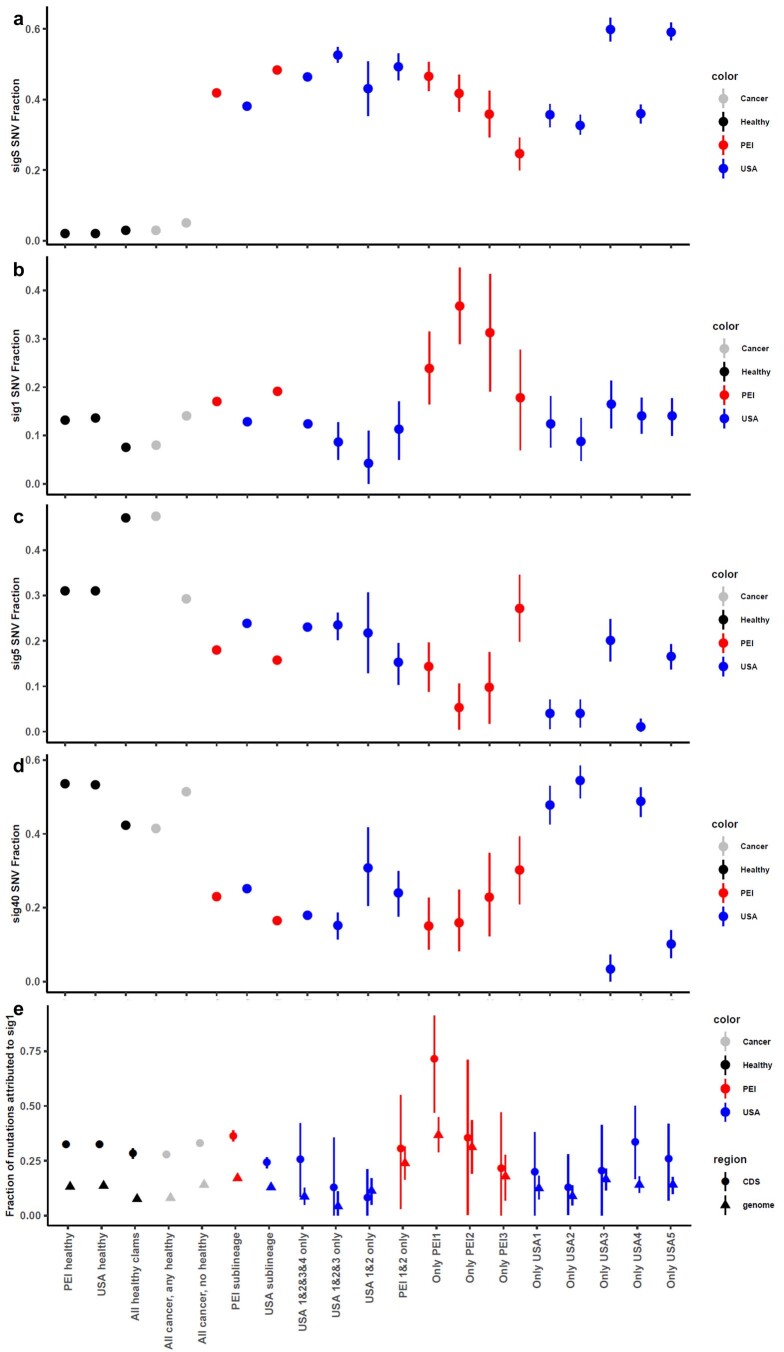
Signature fractions across sample groupings. Plots showing the fraction of genomic SNV fractions attributed to **(a)** signature S, **(b)** signature 1’, **(c)** signature 5’, and **(d)** signature 40’ across healthy and cancer samples, divided and filtered as described in Extended Data Fig. 3, methods, and diagramed in Extended Data Fig. 10. ‘All healthy clams’ refers to SNVs found in all 3 healthy clams in our data set, but not in the reference genome. **(e)** Fraction of mutations attributed to signature 1 across the whole genome (triangles, same data as shown in (b)) is shown compared to the fraction of signature 1 in coding regions alone (CDS, circles). Note that trinucleotide contexts of mutational opportunities are different in coding regions versus the full genome, which was factored into in the signature fitting process. Points indicate fitting estimate, while error bars display 95% confidence intervals of mutation fractions from fitting error of SNVs to the four mutational signatures. Number of total mutations for each SNV set can be found in Extended Data Fig. 3. Source data

By analyzing all identified SNVs and their trinucleotide context, we observed a distinct SNV mutational bias in somatic mutations within both the PEI and USA sub-lineages that was not found in healthy clams (Fig. 2a). These biases are nearly identical in somatic SNVs from both sub-lineages and were also present in more recent mutations, such as SNVs unique to each MarBTN sample (Extended Data Fig. 3a). De novo signature extraction, which deconvolutes mutational biases in their trinucleotide context between samples^28^, yielded four mutational signatures (Extended Data Fig. 3b). Three signatures were found in both healthy clams and MarBTN samples and thus are likely endogenous within the germline of clam genomes. One signature closely resembles COSMIC signature 1 (termed Sig1′), showing a characteristic bias for C > T mutations at CpG sites, which is associated with the deamination of methylated CpGs in humans^29^. Sig1′ represents a greater fraction of mutations in the PEI sub-lineage (Extended Data Fig. 4), which may indicate that PEI has more methylated CpG sites than USA. Sig1′ also represents a greater fraction of mutations in coding regions, fitting previous observations that methylation is elevated in gene regions in bivalves^30^. The other two signatures are ‘flatter’ and less distinctive, most closely resembling COSMIC signatures 5 and 40 (termed Sig5′ and Sig40′), which are both associated with aging in humans^31,32^.

A single signature captured the biases specific to the somatic mutations in MarBTN, termed SigS (Fig. 2b). The closest analog in the COSMIC database of human mutational signatures is signature 9, which shares a T > G bias in A/T trinucleotide contexts^31^. Signature 9 in humans represents mutations induced by polymerase eta during somatic hypermutation and translesion synthesis in humans^31,33^. This may indicate that an error-prone polymerase with similar biases to human polymerase eta is broadly upregulated in cancer or induced due to a high level of DNA lesions during MarBTN replication. In addition to the notable T > G bias in A/T contexts, there is also a notable bias toward C > A mutations compared to healthy clam SNVs, particularly CC > CA and TCT > TAT. Notably, both C > A and T > G mutations have been linked to oxidative DNA damage^34^. Clam hemolymph is strongly hypoxic in late stages of the disease^35^, so this environment may also be contributing to these mutational biases.

### MarBTN is several centuries old

**Extended Data Fig. 5:**
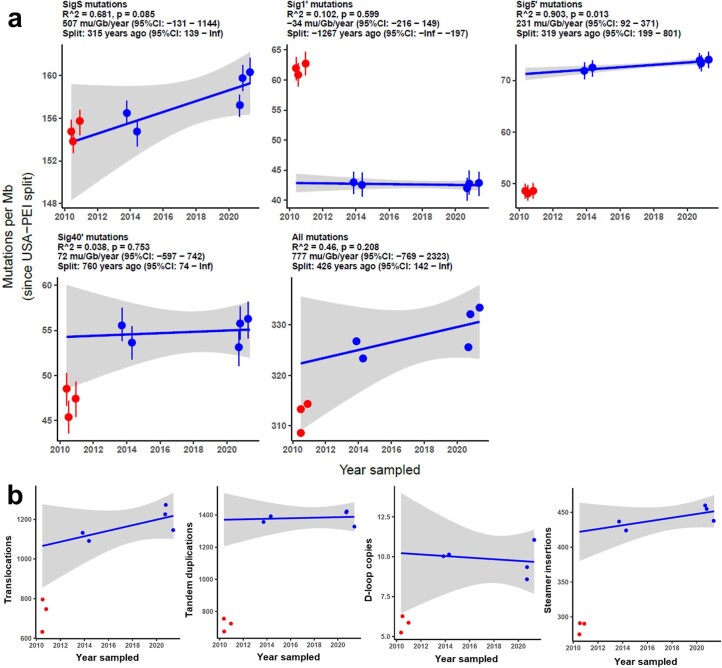
Mutations versus sampling date. **(a**) Mutations attributed to each mutational signature versus sampling date for MarBTN samples. SNVs found in healthy clams, all BTN samples, or LOH regions are excluded prior to analysis to remove founder variants. Results from linear regression of USA samples (n = 5) are shown above each plot, including R squared, p value, mutation rate estimate and the corresponding x-intercept (indicating date the two sub-lineages diverged from one another). PEI samples (n = 3) are included on plots to compare relative mutation counts attributed to each signature but are not included in the linear regression. It is apparent that sig1’mutation counts are higher in PEI, while sig5’ and sig40 mutations are higher in USA. SigS mutations in PEI line up well with the USA sample regression, indicating that sigS mutation rate has stayed stable since the sub-lineages diverged. Points indicate fitting estimate, while error bars indicate 95% confidence interval from signature fitting error. **(b)** Number of translocations and tandem duplications since the divergence of the sub-lineages, copies of the mitochondrial D-loop, and total Steamer insertions per sample, each plotted against sampling date. Linear regression (blue line) and 95% confidence interval (gray) were calculated for the USA samples (n = 5). No regression was statistically significant. No PEI samples (n = 3) fell within 95% confidence intervals of regression lines, indicating the higher mutation counts in USA samples cannot be explained by the later sampling of USA samples. Source data

Signatures 1 and 5 are considered clock-like in humans and other mammals^36,37^ and signature 1 was used to date CTVT’s origin to 4,000–8,500 years before present^12^. We took advantage of the temporal distribution of our USA samples to test whether any signatures were clock-like in MarBTN. We fitted somatic mutations for each sample (SNVs not in other sub-lineages and outside LOH regions) to the four extracted signatures and regressed mutations attributed to each signature against sample collection date (Extended Data Fig. 5a). Sig1′ did not correlate with time, perhaps due to methylation changes affecting CpG > TpG mutation rates and/or inherent differences between clams and mammals. Sig5′ mutations did display a strong correlation with time within the USA samples (Fig. 2c; *P* = 0.013). Assuming the Sig5′ mutation rate has remained steady since USA diverged from PEI, this corresponds to the sub-lineages diverging 319 years ago (95% CI 199–801 years); however, PEI samples have 33% fewer Sig5′ mutations than USA samples, indicating that the Sig5′ mutation rate differs between sub-lineages. SigS mutations also seem to increase with time and although the correlation is not statistically significant within the USA sub-lineage (Fig. 2d; *P* = 0.085), the number of SigS mutations in PEI samples fall within the range predicted by the linear regression of USA samples (Extended Data Fig. 5a). Minimal deviation in the SigS accumulation over time across both sub-lineages, despite their deep divergence, indicates that the mechanism producing SigS mutations is remarkably steady, although the lack of recent PEI samples does not allow us to independently test whether SigS continues to accumulate at the same rate in PEI. Based on the rate calculated from the USA samples, the sub-lineages diverged 315 years ago (95% CI 139–infinity years), in close agreement with our Sig5′ estimate. This estimate lacks an upper bound due to the small number of USA samples and higher deviation of SigS in comparison to Sig5′; however, we can be more confident in the stability of the SigS mutation rate than Sig5′ given the consistency in SigS between the sub-lineages.

As SigS is specific to somatic mutations, we can use it to estimate how many of the mutations shared by all cancers are somatic mutations and therefore estimate how long before the sub-lineage divergence the cancer first arose in the founder clam and began horizontal transmission. SigS contributed roughly half of high-confidence somatic mutations in each sub-lineage but was virtually absent from SNVs in the healthy clam population (Fig. 2e). If we assume that the SigS mutation rate has remained constant since oncogenesis and that the founder clam SNVs have a similar profile of genomic SNVs to those observed in healthy clams, we estimate that 3.1% of heterozygous SNVs found in all cancer samples, but no healthy samples, are somatic mutations attributed to SigS. This corresponds to 108 years by the SigS rate estimate above, for a total cancer age estimate of 423 years (95% CI 187–infinity years) (Fig. 2f), long before the first recorded observations of disseminated neoplasia in soft-shell clams in the 1970s^19,20^.

If we also assume the fraction of SigS somatic mutations has remained constant since oncogenesis, we estimate that, in addition to the 3.1% SigS SNVs estimated above, approximately 3.7% (95% CI 3.4–4.0%) of heterozygous SNVs found in all cancer samples, but no healthy clams, are somatic mutations due to the other three signatures. Combining this estimate (116,765 mutations) with sub-lineage-specific mutations (320,715 and 331,167) we calculate a total somatic SNV estimate of 441 and 452 mutations per Mb for the PEI and USA sub-lineages, respectively. This is a much higher mutation density than that estimated for the <40-year-old DFTD lineages (DFT1, <3.1 mutations per Mb; DFT2, <1.3 mutations per Mb)^15^, but less than the >4,000-year-old CTVT (~867 mutations per Mb from exome data)^12^, showing that mutation density generally scales with age across the small number of characterized transmissible cancer lineages.

### Selection on SNVs is largely neutral

We used the ratio of nonsynonymous to synonymous coding changes (dN:dS) to infer selection acting on coding regions in our sample set. After correcting for mutational opportunities in coding regions, a ratio of one indicates neutral selection, >1 indicates positive selection and <1 indicates negative/purifying selection. We used dNdScv^38^ to determine that the global dN:dS for healthy clam SNVs was 0.454 (95% CI 0.451–0.457), indicating that genes are generally under negative selection in clam genomes, as expected. On a gene-by-gene basis, 70% of intact coding genes (16,222 out of 23,273) in healthy clams have significantly negative dN:dS, whereas 0.4% (88 out of 23,273) are significantly positive. Genes under positive selection in hosts may be those at the host–pathogen interface that are under selection for continued nonsynonymous mutation. In the case of clams, some of these genes may be a response to MarBTN evolution itself, though this hypothesis cannot be tested by the current study.

High-confidence somatic mutations had a global dN:dS of 0.982 (95% CI 0.943–1.024), indicating that MarBTN is largely dominated by neutral selection, reflecting observations in human cancers^39^ and CTVT^12^ (Fig. 2g). We found no genes with a dN:dS ratio significantly <1, indicating that no genes are under significant negative (or purifying) selection, but we did identify five genes with a dN:dS ratio significantly >1, indicating positive selection (Supplementary Table 3). For all five of these genes, nearly all somatic mutations were found in a single sub-lineage. Only one of these genes has a dN:dS ratio above one in healthy clams, suggesting that four of five genes are truly under positive selection in only a single sub-lineage and they are not founder or host clam SNVs. The only characterized gene among the four is a *TEN1*-like gene that is under positive selection in the USA sub-lineage. TEN1 is a component of the CTC1–STN1–TEN1 complex, which plays a crucial role in telomere replication and genome stability^40^.

### Widespread structural mutation

**Fig. 3:**
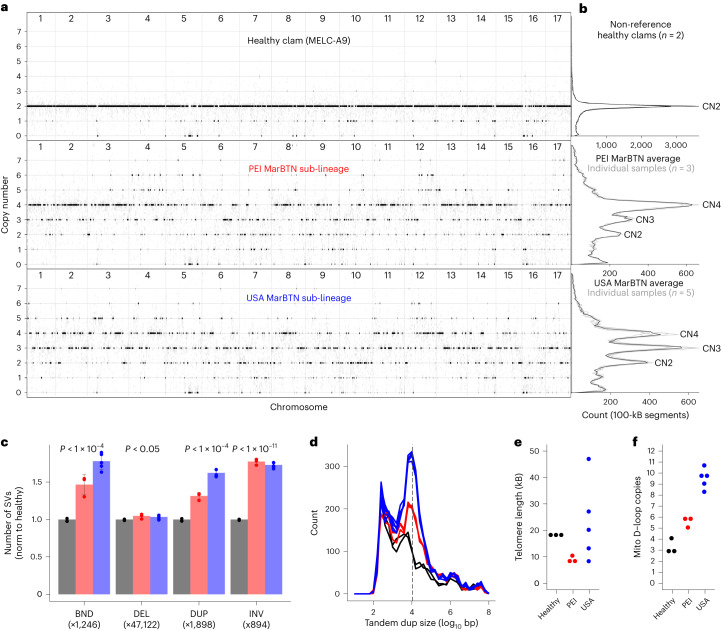
Widespread copy number gain and structural mutation. **a**, Copy number calls across clam genome, rounded to the nearest integer (black) and unrounded (gray) in 100-kB segments. The healthy clam is a representative individual and the MarBTN sub-lineages are averages of each individual sample from that sub-lineage, which were in close agreement. **b**, Summary of copy number states across entire genomes for two non-reference healthy clams and MarBTN sub-lineages. Gray lines display copy number summaries for individual samples within each sub-lineage, which are in close agreement. **c**, Number of SVs in each sample. The reference clam was excluded as one haplotype from that animal was used to build the reference genome and thus does not contain SVs. Values were normalized to the average number of SVs in non-reference healthy clams for each SV type (numbers below SV type labels). *P* values are from two-sided unequal variance *t*-test between MarBTN samples (*n* = 8) and non-reference healthy clams (*n* = 2). Exact *P* values are 1.9 × 10^−5^, 2.9 × 10^−2^, 1.0 × 10^−5^ and 8.0 × 10^−11^, respectively. Labels follow DELLY abbreviations of SV types: BND, translocations; DEL, deletions; DUP, tandem duplications; INV, inversions. Bars indicate means and error bars indicate s.d. **d**, Size distribution of tandem duplications in each non-reference sample. Dashed line indicates 11 kB. **e**, Telomere length estimated by TelSeq for each sample. **f**, Tandem duplicate copies of the mitochondrial D-loop region per sample. Healthy clams are black, MarBTN from PEI are red and MarBTN samples from USA are blue. Source data

Polyploidy has been described in disseminated neoplasia in several bivalve species^27,41^. In *M.* *arenaria*, disseminated neoplasia cells have approximately double the chromosome count and genome content of healthy clam cells^24^. Given the discovery that these cells are of clonal origin^6^, we had hypothesized that a full genome duplication occurred early in the cancer’s evolution and that most of the MarBTN genome should be 4N. To test this theory, we called copy number states across each non-reference sample genome based on read depth (Fig. 3a). As expected, both healthy clams were 2N across nearly the entire genome (Fig. 3b). Notably, MarBTN samples displayed a wide variety of copy number states.

**Extended Data Fig. 6:**
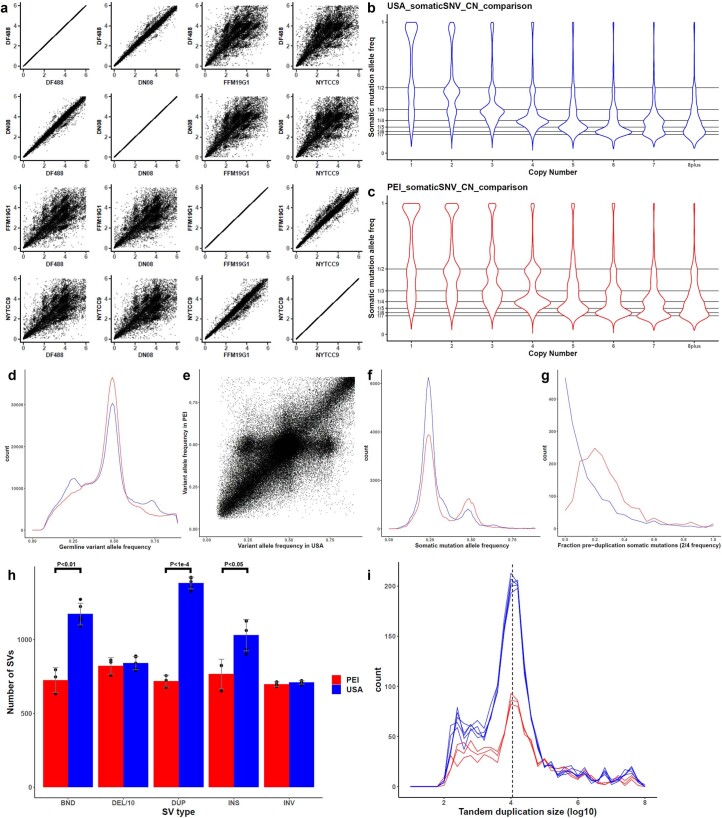
Copy number and structural alteration characterization. **(a**) We called copy number across the genome in 100-kB chunks for each sample individually. Here we plot pairwise comparisons of the copy number call for each 100-kB chunk between two representative PEI BTN samples (DN08 and DF488) and two representative USA BTN samples (FFM19G1 and NYTC-C9: notably, the two most distantly related USA samples). There is a close correlation (R2 > 0.94) within sub-lineages (DN08 vs DF488, FFM19G1 vs NYTC-C9) and a weaker correlation (R2 = 0.53–0.56) when comparing between sub-lineages (DN08 or DF488 vs FFM19G1 or NYTC-C9). Copy number differences between samples can be seen here as denser groupings of points around integer values that deviate from equal values along the diagonal. Variant allele frequencies of all high confidence somatic mutations were calculated separately for BTN from **(b)** USA) and **(c)** PEI. Violin plots show probability densities of allele frequencies of high confidence somatic mutations, divided into portions of the genome called at each copy number. The peak allele frequency in each case is distributed around the expected value of 1/copy number. In addition to the main, expected peaks for each copy number, in some cases, additional peaks can be seen that indicate somatic mutations prior to copy number gain (for example VAF of 0.5 in regions with CN4 that could be due to mutation followed by duplication of the region). Some minor peaks also indicate possible errors in copy number calling or allele frequency counting (e,g, VAF of 0.5 in CN3 regions). These errors could be due to lower read mapping due in polymorphic region, errors caused by repeat regions, regions spanning a CN breakpoint, among other possibilities. **(d)** Distribution of variant allele frequencies for founder germline variants (found in all cancers and at least one healthy sample) in USA (blue) and PEI (red) sub-lineage, restricted to regions that are CN4 in both sub-lineages. **(e)** A random subset of 100,000 germline variants plotted as a scatter plot. Alleles at 1/4 and 3/4 in the USA sub-lineage are incongruent with a simple CN2 > CN4 duplication. **(f)** Distribution of variant allele frequencies for high confidence somatic mutations, restricted to regions that are CN4 in both sub-lineages, showing a higher proportion of 2/4 mutations (pre-duplication SNVs) in PEI than USA. **(g)** The genome was subdivided into 100-kb segments (as done for copy number analysis), and for all shared CN4 segments the plot shows the fraction of mutations in each 100kB segment that were at 2/4 frequency compared to the total amount of 2/4 and 1/4 SNVs, corresponding to mutations occurring before or after duplication of the allele, respectively. While the USA distribution peaks at 0, indicating most 100kB segments duplicated before or shortly after the USA-PEI sub-lineage split, with a low rate of duplications occurring after that time, the distribution for PEI centers around 0.2, indicating that one-fifth of mutations occurred between the USA-PEI sub-lineage split and duplication of the corresponding regions, suggesting a burst of duplications at some point in the PEI sub-lineage. **(h)** Number of called SVs of each type that are unique to each sub-lineage were calculated by removing SVs found in any healthy clams or in any BTN samples from the other sub-lineage. Dots represent individual samples, bars summarize averages for each group, and error bars indicate standard deviation. P-values are from two-sided unpaired unequal variance t-test between PEI BTN samples (n = 3) and USA BTN samples (n = 5). Exact values are 1.8e-3, 6.6e-1, 1.0e-5, 1.9e-2, and 3.6e-1 respectively. Labels follow delly abbreviations of SV types: BND = translocations, DEL = deletions, DUP = tandem duplications, INS = small insertions, INV = Inversions. Deletion counts were much higher than other SV types, so were divided by 10 in (B) for visualization (‘DEL/10’). **(i)** Size distribution of tandem duplications in each sample, after removing SVs found in any healthy clams or in any BTN samples from the other sub-lineage. Dashed line indicates 11 kB. Source data

PEI samples were predominantly 4N with substantial 3N and 2N portions, whereas USA samples were more evenly distributed between 4N, 3N and 2N (Fig. 3b). Copy number calls in cancer samples displayed close agreement within sub-lineages (*R*^2^ > 0.94). There was a positive correlation between copy number calls between the two sub-lineages, but large differences could be observed suggesting that copy number changes have occurred since sub-lineage divergence (*R*^2^ = 0.53–0.56) (Extended Data Fig. 6a). Variant allele frequencies (VAFs) for high-confidence somatic mutations largely support copy number calls (Extended Data Fig. 6b,c), with some off-target VAF peaks, most notably in the lower copy number regions (<3N), indicating that some of these regions have higher copy numbers than called through this method but seemed lower likely due to reduced read mapping in polymorphic genome regions.

To estimate timing of duplication events we looked at VAF in regions called CN4 across both sub-lineages (14% of the genome; Extended Data Fig. 6d–g). While the majority of founder variants were distributed around a VAF of 0.5 (2 of 4 alleles) in both sub-lineages, as expected for a CN2 > CN4 duplication, USA also had VAF distributions around 0.25 and 0.75 (1 of 4 and 3 of 4 alleles) that were absent in PEI, indicative of CN2 > CN3 > CN4 duplication where not all haplotypes duplicated evenly. Additionally, we observe more 2 of 4 high-confidence somatic mutations in PEI than USA, indicative of later duplication events. The fraction of 2 of 4 somatic mutations in the USA sub-lineage was low in nearly all CN4 segments of the genome, indicating most segments duplicated before or shortly after the USA–PEI sub-lineage split, with a low rate of duplications occurring after that time. In contrast, many segments in PEI sub-lineage have around 20% of the somatic mutations at 2 of 4 alleles, suggesting a burst of duplications at some point after the USA–PEI sub-lineage split. Overall, these frequencies indicate the USA and PEI sub-lineages arrived at CN4 largely via independent duplication events, rather than the assumed single whole-genome duplication and that duplication events have occurred at multiple points throughout MarBTN evolution.

Many mid-chromosome breakpoints were apparent in the copy number calls, indicating that the MarBTN genome has likely undergone widespread structural alterations in addition to whole-chromosome and within-chromosome copy number gain. We are unable to resolve the structure of the MarBTN genome with the short sequence reads in our current dataset but were able to call likely structural variants (SVs) from split reads. Relative to non-reference healthy clams, MarBTN samples had a significantly higher number of deletions, inversions, tandem duplications and inter-chromosomal translocations, indicating substantial somatic structural alterations (Fig. 3c).

Comparing likely somatic SVs specific to each sub-lineage, USA samples had significantly more translocations and tandem duplications than PEI (Extended Data Fig. 6h). Median somatic tandem duplication sizes displayed a distinct distribution around a mode of ~11 kB (Fig. 3d and Extended Data Fig. 6i). In human cancers, tandem duplication phenotypes of this same size distribution are thought to be driven by the loss of *TP53* and *BRCA1* (ref. ^42^), indicating that a parallel mutational process may be influencing the observed genome instability in MarBTN and more active in the USA sub-lineage.

Maintenance of telomere length is a requirement for an immortalized cell line such as MarBTN and would be necessary for long-term survival. We estimated telomere lengths for each sample and found them to be highly variable within the USA sub-lineage (8–47 kB), whereas they were short but relatively stable within the PEI sub-lineage (8–11 kB) compared to healthy clams (18–19 kB) (Fig. 3e). Variable telomere lengths in the USA sub-lineage may relate to the *TEN1*-like gene that is under positive selection in that sub-lineage, as the CTC1–STN1–TEN1 complex inhibits telomerase and is involved in telomere length homeostasis^40^.

### Mitochondrial genome evolution

**Extended Data Fig. 7:**
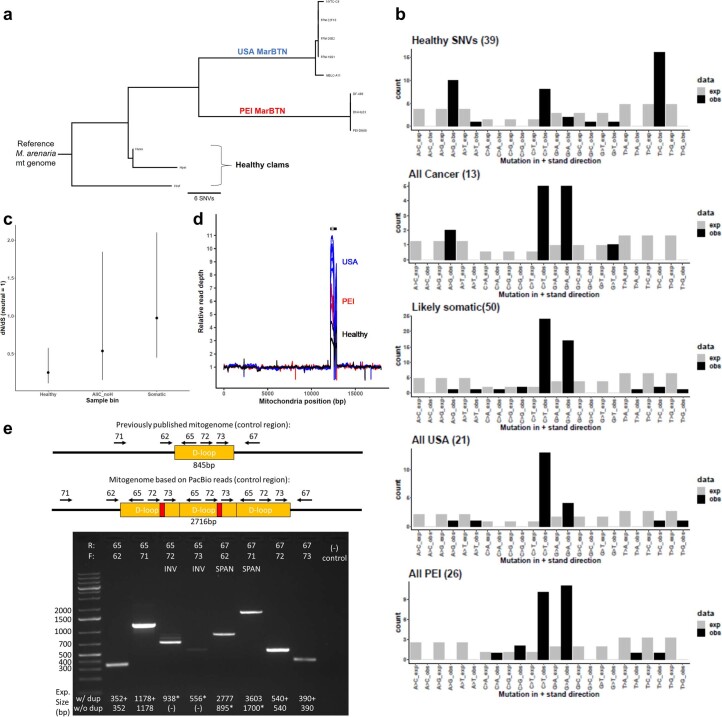
Mitochondrial mutations in MarBTN. **(**a) Neighbor joining tree built from variants called in all samples (170 SNVs) against the previously published *M. arenaria* reference mitogenome (excluding the repeated region). Bootstrap values in support of each clade are included on the preceding branch (bootstraps under 50 are not shown). The phylogenetic relationship generally reflects that built from genomic SNVs (that is, monophyletic MarBTN group with separate USA and PEI sub-lineages). The phylogeny within the USA sub-lineage deviates from that built from the nuclear genome, but only three SNVs are variable within the USA sub-lineage: one SNV unique to NYTC-C9 and two SNVs unique to MELC-A11. This causes the other samples to cluster more often with NYTC-C9 due to only one difference (versus two versus MELC-A11), but this relationship is still compatible with the USA branch structure from the nuclear phylogeny. **(b)** Observed SNVs (black) compared with expected counts estimated from nucleotide frequencies of the *M. arenaria* mitogenome and assuming equal mutation probability. This calculation was not collapsed to the usual 6 mutation types due to the imbalance of nucleotides in mitochondrial genomes (unequal frequencies of G/C and A/T). Likely somatic refers to SNVs found in a subset of BTN samples, while All USA and All PEI refer to SNVs found in all individuals from that sub-lineage, but not the other sub-lineage. **(c)** dN/dS ratios, where a ratio of 1 indicates neutrality, were calculated for mitochondrial SNVs found in healthy clams (n = 39), all BTN samples but not healthy clams (n = 13), and likely somatic mutations (n = 50). Error bars indicate 95% confidence intervals as estimated by dndscv and are quite large, due to the low number of mitochondrial SNVs. **(d)** Read depth across the mitochondrial genome for healthy clams (black), PEI MarBTN (red) and USA MarBTN (blue), normalized to mean depth outside D-loop. Bars above indicate the D-loop region (12,164–12,870 bp, black) and the region used to estimate duplicated region copy number (12,300–12,500 bp, gray), as shown in Fig. 3f. **(e)** Schematic (not to scale) of the control region of the *M. arenaria* control region in the previously published mitogenome with a single d-loop copy (top) versus the proposed mitochondrial genome with three d-loop copies and G-rich insertions (middle) with accompanying PCR results (bottom). Primer pair combinations are listed along top of gel and expected sizes are listed along bottom, molecular weights are in bp. Amplicon sizes from primers spanning the D-loop (67 with 62/71) support a single copy of the D-loop. However, we suspect this is a result of recombination and selection for the smaller product and loss of the G-rich insertions. Inverse PCR with outward-facing primers (65 with 72/72) indicates a tandem duplication allowing outward-facing primers to amplify. The inverse primers spanning the G-rich insertion (65 with 72) has a dim band at expected size, but two brighter bands at smaller sizes. PCR was run once. Source data

A tree built from pairwise mitochondrial SNV differences between samples reflects a similar phylogeny to that built from genomic SNVs (Extended Data Fig. 7a). This indicates no evidence of mitochondrial uptake or recombination with host mitochondria, which has been observed in other transmissible cancers^8,43,44^. Transitions were highly overrepresented in both healthy and cancer samples, with C > T mutations composing 41 of 50 likely somatic mutations (Extended Data Fig. 7b). Somatic mutations resulted in missense mutations in at least 10 of the 12 mitochondrial genes, and the genes seem to be under relaxed selection, with dN:dS ratios of 0.97 (95% CI 0.45–2.1) versus 0.26 (95% CI 0.11–0.58) for SNVs in healthy clams (Extended Data Fig. 7c).

When aligned to the published *M.* *arenaria* mitochondrial genome^45^, short read sequences from all MarBTN and healthy samples display increased coverage across the mitochondrial D-loop (Extended Data Fig. 7d), indicating the region is multi-copy. The D-loop is part of the non-coding control region of the mitochondrial genome and is the origin of both replication and transcription. We resolved this region with PacBio long reads from the healthy reference clam, revealing three copies in tandem. Two of the copies contain a 236-bp insertion not found in the published mitochondrial genome. The insert includes an 80-bp region with 70% guanine content, likely complicating previous PCR-based efforts to resolve it. Altogether, the observed copies extend the D-loop region of the reference clam genome from 845 bp to 2,727 bp and the full mitochondrial genome to 19,815 bp.

Read coverage of the D-loop region suggest that there have been additional somatic tandem duplications in the MarBTN mitogenome. While read coverage indicates 3–4 copies in the non-reference healthy clams, PEI MarBTN samples have 5–6 copies and USA MarBTN samples have 8–11 (Fig. 3f). These somatic tandem duplications likely arose via replication errors and the trend toward increased copies in cancer suggests that they may be under selection. Selection can act on the level of the mitogenome itself, giving it a replicative advantage over other mitogenomes (as hypothesized for CTVT) or on the level of the cancer cell, if this duplication provides cancer cells a replicative advantage over others. Notably, the mitogenome site suspected to be under selection during repeated mitochondrial capture in CTVT is in the control region^44^, the same region we see amplified in MarBTN.

### Transposable element mobilization

**Fig. 4:**
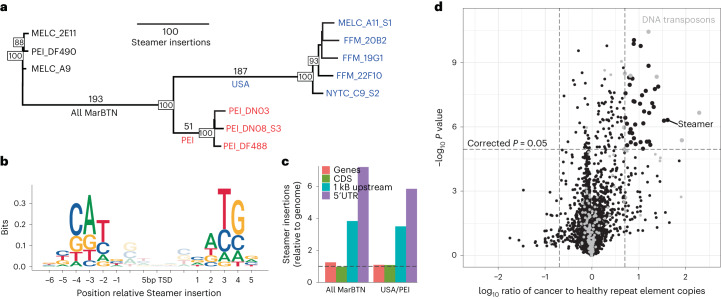
Somatic expansions of Steamer and other TEs. **a**, Phylogeny of all samples built from pairwise differences of Steamer insertion sites, colored by healthy (black), USA MarBTN (blue) and PEI MarBTN (red). Numbers along branches indicate the number of insertions unique to and shared by individuals in that clade, numbers on nodes indicate bootstrap support, with bootstrap values below 75 not shown. **b**, Logo plot of insertion bias relative to the 5-bp target site duplication (TSD) of all Steamer insertions, normalized by nucleotide content of the genome. **c**, Steamer insertion probability in annotated genome regions, normalized by read mapping rates and relative to full genome. Displayed for insertions found in all MarBTN samples but no healthy clams and unique to each sub-lineage but shared by all individual in that sub-lineage. Dashed line indicates expectation given random insertions. **d**, Volcano plot comparing copy number of all repeat elements in MarBTN and healthy clam samples by two-sided unequal variance t-test. Dashed lines correspond to significance threshold (*P* = 0.05, Bonferroni-corrected) and fivefold differences. Elements annotated as DNA transposons are marked in gray. Source data

MarBTN is known to contain the LTR retrotransposon, Steamer, at a much higher copy number than healthy clams, indicating likely somatic expansion^46^. To test whether Steamer activity is ongoing we identified Steamer insertion sites using split reads spanning Steamer and the reference genome. Only 5–11 sites were found in each healthy sample, versus 275–460 sites in each cancer sample. A total of 193 sites are shared by all cancer samples, indicating that Steamer expansion likely began early in the cancer’s evolution, whereas sub-lineage-specific Steamer integrations indicate that Steamer has continued to replicate somatically in the MarBTN genome (Fig. 4a); however, Steamer has generated more insertions within the USA sub-lineage (*n* = 248) than the PEI sub-lineage (*n* = 64), indicating the regulatory environments of the sub-lineages have not remained stable since they diverged.

**Extended Data Fig. 8:**
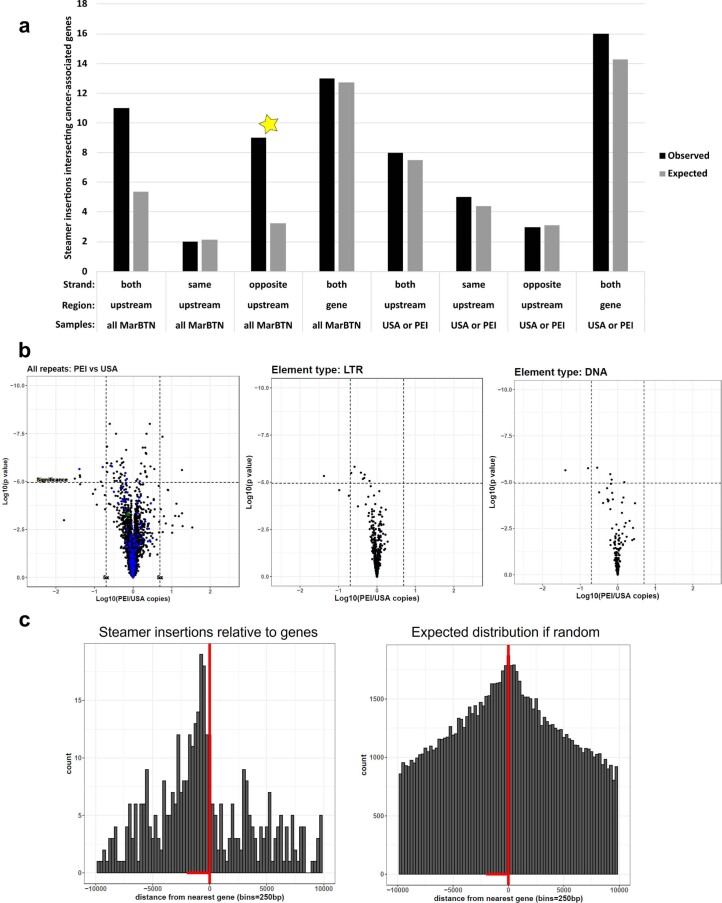
Transposable element activity in MarBTN. **(a**) We conducted a BLASTP search for the 729 cancer-associated genes in the COSMIC database and found hits in 5,430 of the 38,609 predicted M. arenaria genes (14%). If there is not selection for insertion near these genes, we would expect 14% of Steamer insertions with a M. arenaria gene to intersect with these genes. We counted the number of steamer insertions in genes (‘gene’) and in the 2 kB upstream genes (‘upstream’) for early steamer insertions in the lineage trunk (‘all MarBTN’) and after the divergence of the sub-lineages (‘USA or PEI’). We plotted these counts (black) against that expected by chance (gray). Counts match expected closely for late insertions (in only the USA or PEI sub-lineage – right side of plot), either upstream genes or within them, but were higher than expected for early insertions. We further divided upstream insertions by whether the steamer insertion was in the same strand/direction as the gene or opposite, to compare with counts regardless of directionality (‘both’). The early insertion bias to insert upstream cosmic genes can be fully explained by a bias to insert in the opposite strand (yellow star), here with 9/23 (39%) of the genes being cancer associated (would expect 3/23: Chi-squared test, Bonferroni-corrected p value = 0.004). **(b)** Volcano plots showing estimated copy number of each TE, comparing copy number from PEI MarBTN with USA MarBNT for all TE types (left), LTR elements (middle), and DNA transposons (right), compared by two-sided unequal variance t-test. TEs more highly amplified in PEI MarBTN are to the right and TEs amplified more highly in USA MarBTN are to the left. Dashed lines correspond to significance threshold (p = 0.05, Bonferroni-corrected) and 5-fold differences. DNA transposons are labeled in blue and Steamer is labeled in green. Eight LTR retrotransposons and five DNA transposons are significantly amplified in the USA sub-lineage compared to the PEI sub-lineage, while no identified LTR retrotransposons and a single DNA transposon TEs are significantly amplified in the PEI sub-lineage compared to the USA sub-lineage. **(c)** Left histogram showing the distance to nearest gene for Steamer insertions found in any cancer sample (n = 550). If an insertion was within an annotated gene, the distance to the next nearest insertion was used. 0 (vertical red line) corresponds to the first or last nucleotide of the annotated gene for when the insertion is upstream (negative) or downstream (positive) relative to the gene, respectively. Horizontal red segment highlights 2 kB upstream genes with elevated Steamer insertions. Right histogram shows a distribution of randomly generated insertion sites (n = 224,134) based off the observed read mapping in the genome assuming insertions are random. Source data

We also observed strong biases for Steamer to insert at specific genomic sequences. Steamer has a palindromic bias for NATG outside the five bp target site duplication (CATNnnnnnNATG), inserting at these locations 45× more frequently than expected by chance (Fig. 4b). Steamer was also >3× more likely to insert within 1,000 bp upstream of genes than would be expected by chance (Fig. 4c). We also observed early Steamer insertions (those found in all MarBTN samples) upstream of cancer-associated orthologs more often than expected by chance in the reverse but not the forward orientation (Extended Data Fig. 8a and Supplementary Table 4). This bias, which could indicate either an insertion preference for those locations or a selective advantage to MarBTN cells, was associated with those insertions.

We further investigated whether other transposable elements (TEs) in addition to Steamer have expanded somatically by identifying a library of repeat sequences (putative TEs) found in clam genomes and counting the copy number of each TE type in each sample. Forty-five TEs were present at a significantly higher copy number in cancer samples relative to healthy clams after removing TEs with fewer than fivefold differences (Fig. 4d). TEs annotated as DNA transposons were enriched in this dataset (8 of 45, 17.8%) compared to the total TE library (171 of 4,471, 3.8%), indicating this TE type may have been particularly successful in somatically expanding its copy number in MarBTN. LTR retrotransposons (such as Steamer) seem to have had more success in the USA versus PEI sub-lineage. Thirty-six TEs have significantly more copies in the USA sub-lineage than PEI and eight of those are LTR retrotransposons, compared to 0 LTR retrotransposons out of 20 of those more highly expanded in PEI (Extended Data Fig. 8b). Reduced copy numbers of LTR retrotransposons and other TEs in the PEI sub-lineage could be linked to the increased methylation indicated by mutational signature analysis, as methylation is thought to repress TE mobilization^30,47^. Our finding of widespread increases in TE copy numbers alongside structural mutations indicate general genome instability of the MarBTN lineage and provides further evidence of a higher rate of certain mutation types in the USA sub-lineage, which cannot be explained by the temporal distribution of the samples alone (Extended Data Fig. 5b).

### MarBTN gene expression

**Fig. 5:**
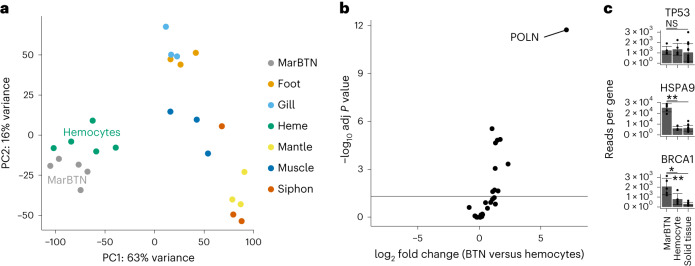
Expression indicates hemocyte origin and possible mutagenic pathways in MarBTN. **a**, Principal-component analysis of normalized expression across all genes, with PC1 separating MarBTN and hemocytes from all other tissues. **b**, Volcano plot of expression of polymerase genes (*n* = 28 genes) for MarBTN (*n* = 5 isolates) compared to hemocytes (*n* = 5 clams). **c**, Normalized expression, in reads per gene, of TP53, HSPA9 (mortalin) and BRCA1 for MarBTN (*n* = 5 isolates), hemocytes (*n* = 5 clams) and non-hemocyte tissues (*n* = 15: 5 tissues for three clams). Error bars display standard deviation, differential expression comparison results from Wald test displayed as *P < 0.05; **P < 1×10^−5^; NS, not significant. Exact *P* values, adjusted for multiple comparisons, are 5.5 × 10^−1^, 6.8 × 10^−1^, 8.4 × 10^−8^, 5.0 × 10^−7^, 3.3 × 10^−2^ and 1.6 × 10^−9^, respectively. Source data

**Extended Data Fig. 9:**
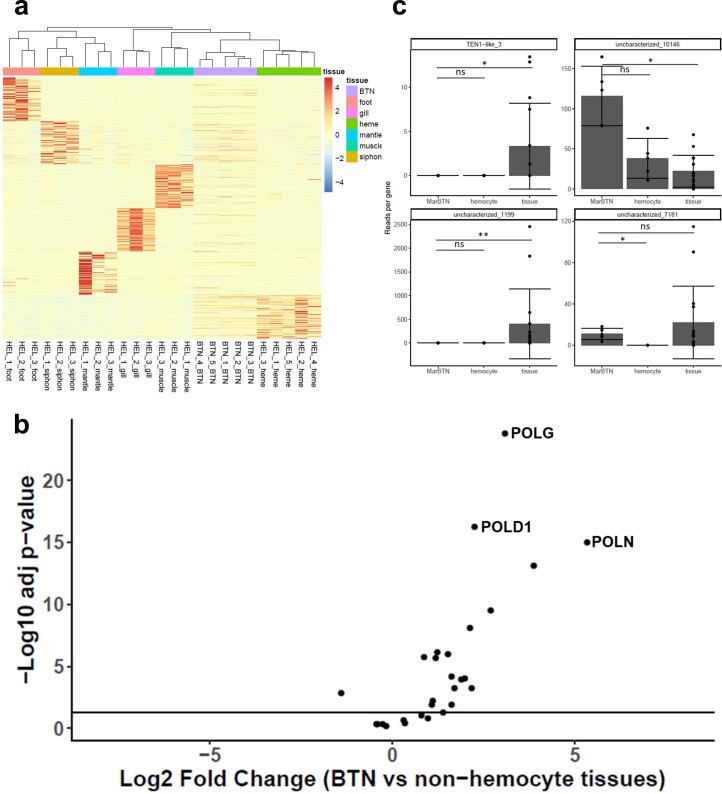
Differential expression results. **(a**) Hierarchical clustering of all RNA sequenced samples by the expression of the top 100 most significant genes expressed in each specific healthy tissue relative to all other tissues, with heatmap of normalized relative gene expression for each gene. MarBTN (BTN) clusters most closely with hemocytes (heme), supporting principal-component analysis results. **(b)** Volcano plot of polymerase genes expression (n = 28) for MarBTN (n = 5) compared with non-hemocyte tissues (n = 15: 5 tissues for 3 clams). **(c)** Normalized expression, in reads per gene, of four genes with detectable positive dN/dS for MarBTN (n = 5), hemocytes (n = 5), and non-hemocyte tissues (n = 15: 5 tissues for 3 clams). Bars display mean, error bars display standard deviation, and differential expression comparison results displayed as * = p<0.01, ** = p<1e-7, ns = not significant. Exact p-values are 9.6e-1, 3.7e-2, 1.8e-1, 8.1e-3, 7.4e-1, 1.5e-8, 6.0e-3 and 3.1e-1 respectively. Source data

To investigate the role of genes implicated in MarBTN evolution we sequenced RNA from a new set of five MarBTN isolates from the USA sub-lineage, six tissues (hemocytes, foot, gill, adductor muscle, mantle and siphon) across three healthy clams and hemocytes from an additional two clams (Supplementary Table 5). Both principal-component analysis and hierarchical clustering clearly separate MarBTN and hemocytes from all solid tissue samples (Fig. 5a and Extended Data Fig. 9a), indicating MarBTN likely originated as a hemocyte. This origin has been hypothesized due to MarBTN being most obviously detectable in the hemolymph^6,48^, but had not previously been tested.

MarBTN-specific SigS resembles an error-prone polymerase signature in humans, so we first compared the expression of the 28 polymerase genes identified in the clam genome. We observed widespread upregulation across polymerases in MarBTN (Fig. 5b and Extended Data Fig. 9b), likely facilitating increased cellular replication and/or DNA damage repair. The most highly upregulated polymerase is homologous to polymerase Nu (*POLN*), a very low fidelity polymerase that plays a role in translesion synthesis and cross-link repair by homologous recombination^49,50^. Polymerase Nu frequently mis-incorporates dT opposite a template dG in humans^51,52^, a bias which does not match SigS; however, given the distance between bivalves and humans, it is possible that this polymerase introduces different biases in clams and is in part responsible for the observed SigS biases and/or genome instability.

We next looked at the expression of four genes under putative positive selection as identified by dN:dS (Extended Data Fig. 9c). Positive selection in cancer can indicate repeated selection for either loss-of-function or gain-of-function mutations. Two genes were not expressed in MarBTN, including the *TEN1*-like gene, indicating a potential loss of function, whereas two genes were upregulated in MarBTN versus healthy hemocytes, indicating a potential gain of function.

Finally, we investigated genes implicated by the distinct ~11 kB tandem duplication phenotype; *TP53* and *BRCA1*. Previous work identified the deactivation of p53 via cytoplasmic sequestration by overexpressed mortalin^53^, so we investigated the expression of genes homologous to *TP53* and mortalin-encoding *HSPA9* (Fig. 5c). Indeed, whereas *TP53* had no nonsynonymous MarBTN mutations and was not differentially regulated, *HSPA9* was significantly upregulated in MarBTN samples, supporting the proposed model of inactivation by mortalin sequestration. Similarly, clam *BRCA1* homolog has no obvious loss of function mutations (three missense SNVs were observed in all MarBTN samples, which do not correspond to known loss-of-function mutations and could be either somatic mutations or inherited founder variants). The tandem duplicator phenotype was reported to be strongly associated with loss of function of BRCA1^42^ in humans, but, in MarBTN, BRCA1 was upregulated (Fig. 5c). We speculate that either (1) BRCA1 is rendered non-functional by some other mechanism (similar to p53); (2) it is functionally overwhelmed by genome instability over the long timescale of this cancer lineage, resulting in a similar phenotype to loss of function; and/or (3) a different pathway in bivalves is responsible for the tandem duplication phenotype; although we are unable to test these hypotheses in this study. Overall, MarBTN gene expression illuminates possible mechanisms behind the lineage’s observed genome instability, though much remains unknown about the forces generating and tolerating such widespread genomic alterations.

## Discussion

Our genome analyses reveal a diverse set of somatic mutations occurring in MarBTN, with continued accumulation of SNVs and widespread structural mutations indicative of genome instability. It is unclear whether these mutations have consistently occurred over time or have been generated in multiple punctuated chromothripsis-like events, but the continued accumulation of these changes between the sub-lineages shows that this instability was not confined to a single ancestral event. Genomic studies of the dog and Tasmanian devil transmissible cancers have shown contrastingly stable genomes, remaining predominantly diploid, despite thousands of years of evolution in the case of CTVT^11,54^. Polyploidy has been reported in other BTN lineages in other bivalves^27^, indicating that genome instability may be a common driver mechanism or a tolerated by-product of conserved processes in BTN evolution. Of note, while there is ongoing instability in both sub-lineages, we observed differences in the number of structural mutations, duplication timing, telomere length and TE amplification between the two sub-lineages, suggesting that genome instability or mutation tolerance may have changed over time in MarBTN after the sub-lineages diverged. These changes in fundamental mutational mechanisms observed in distinct sub-lineages post-divergence highlight the fact that oncogenesis is not a single event, but an ongoing evolutionary process.

In contrast to the above unstable and variable structural processes, we observed a pattern of consistent single-nucleotide mutation biases in both sub-lineages. Most notable is the distinct profile and consistent accumulation of mutational signature S. We hypothesize that this signature is due in part to an upregulated error-prone polymerase and that its consistent accumulation may be due to consistent MarBTN replication rates over time, as seen in human somatic cells with defective proofreading polymerases^55^ or due to continual damage of chromosomal DNA and its repair using translesion synthesis. Both SigS and Sig5′ (analogous to the clock-like signature five in humans) generate consistent age estimates for the most recent common ancestor of our sample set and estimate the cancer is at least 200 years old, though uncertainty in the calculated mutation rates means the actual age of the cancer could be far greater. This indicates that MarBTN is likely an intermediate age compared to DFTD (<40 years^16^) and CTVT (4,000–8,500 years^12^).

We observed that the MarBTN genome is largely dominated by neutral selection, reflecting observations in human cancers^39^ and CTVT^12^, with a few notable genes under positive selection in a single sub-lineage, which may reflect selection for repeated mutations involved in critical oncogenic processes; however, we also note that selection is not simply relevant at the level of the cancer cell, but also on the level of the gene (as seen in MarBTN TE expansions), mitochondria (as seen in CTVT horizontal transfer^44^ and MarBTN mitogenome expansion) and hosts (as seen in DFTD^56^). Further analysis of MarBTN and other cancers will help us to understand how these selective forces interact to influence cancer evolution and perhaps how we can manipulate those forces to our advantage to combat conventional and transmissible cancers.

Our analysis of the MarBTN genome is presented simultaneously to an independent analysis of two lineages in the common cockle (*Cerastoderma* *edule*) or CedBTN, by Bruzos and colleagues^57^. CedBTN infection presents as a similar leukemia-like disseminated neoplasia phenotype to MarBTN and gene expression points toward a hemocyte origin for BTN in both species. The CedBTN genomes display signatures of ongoing instability such as MarBTN, supporting the hypothesis that genome instability is a common feature of BTN evolution and confirming that long-term survival of a cancer lineage can be maintained despite remarkably widespread and continued genome rearrangement. This level of instability might be expected to lead to an error catastrophe^58^, yet these cancers have continued to replicate for centuries, changing our understanding of what is possible in cancer evolution. Tandem duplications in the mitochondrial control region were also observed in both studies and may represent convergent evolution driven by the same selective mechanisms. Similar tandem duplications in the D-loop have also been observed in human cancers^59,60^, though the functional consequences of these mutations remain unclear. Repeated expansion of this region in independent BTN lineages, along with their long history of coevolution with their hosts, make BTNs unique model systems for the understanding of the functional significance of mitogenome mutations on cancer cell growth and the potential for selfish selection at the level of the mitogenome in cancer.

In contrast, our finding of a distinctive polymerase-associated mutational signature, evidence of positive selection, variable telomere length and amplification of the Steamer retrotransposon and other TEs may be unique features of the BTN in clams. We find no evidence of mitochondrial genome transfer events or host co-infection by multiple clones as observed in cockles, though this may be due to the smaller sample size of our study and the low level of polymorphisms in mitochondrial DNA in soft-shell clams. Given the apparent abundance of BTNs, continuing to analyze BTN lineages in other species may reveal both common and unique pathways that have allowed these cancers to repeatedly circumvent new host immune systems and spread through host populations as contagious cancers. These cancers therefore provide unique models for the understanding of cancer evolution and exemplify what genomic changes are possible in long-lived cancers evolving together with their hosts.

## Methods

### *M.arenaria* genome assembly

#### Reference animal collection and sequencing

Due to the high rate of heterozygosity in bivalves, a single clam was chosen to be the source of all DNA used in the generation of the reference genome and a diploid phased assembly strategy was used. The reference animal (MELC-2E11, 62 mm shell length; Fig. 1b) was collected from Larrabee Cove, Machiasport, Maine, USA in June 2018 and shipped to the Pacific Northwest Research Institute laboratories. Hemolymph was drawn from the pericardial sinus using a 0.5 in 26-gauge needle on a 3-ml syringe and it was checked for the presence of MarBTN through morphological analysis (Fig. 1c) and with a sensitive cancer-specific qPCR assay^17^, with no evidence of detectible BTN. Examination of the gonad region revealed the presence of eggs, showing that this individual was female.

High molecular weight (HMW) DNA, used for PacBio sequencing, was extracted from snap-frozen mantle tissue using a modified CTAB extraction protocol (adapted from elswhere^61^). HMW DNA was also extracted using the MagAttract HMW DNA kit (QIAGEN) and used for 10x Chromium sequencing. RNA was extracted from six tissues, frozen at −80 °C in RNAlater (Invitrogen) and RNA sequenced (1, mantle; 2, foot; 3, siphon; 5, adductor muscle; 6, gills; and 7, hemocytes). Extraction details are outlined in the Supplementary Note.

#### Diploid assembly, Hi-C scaffolding, gap-filling and polishing

HMW DNA extracted using the CTAB protocol was sequenced using the PacBio core facility at the University of Washington Department of Genome Sciences. Due to the high heterozygosity in bivalve genomes, the FALCON-Unzip pipeline was run to generate a diploid-aware de novo assembly. The resulting assembly can be expressed as either as two pseudo-haploid reference genomes or as a primary assembly with alternate ‘haplotigs’ in genomic regions where the two copies of the diploid genome in the reference individual differ. The purge_haplotigs pipeline^62^ was used to remove pairs of contigs that were called as separate primary contigs by FALCON-Unzip but which are more likely to be alternate alleles, generating a new curated assembly (Mar.3.2.3_curated.FALC.fasta).

Chromatin conformation capture data was generated using a Phase Genomics Proximo Hi-C Animal kit, which is a commercially available version of the Hi-C protocol^63^ and Phase Genomics’ standard Hi-C alignment protocol^64^.

PBJelly was run to gap-fill the scaffolded assembly using pbsuite^65^ (v.15.8.24, slightly modified; https://github.com/esrice/PBJelly) using blasr (v.5.1) and networkx (v.2.2) with Python v.2.7, with the protocol file Protocol_MELC.xml.

We used a phase-aware polishing strategy, modified from the pipeline described in the Vertebrate Genome Project (https://github.com/VGP/vgp-assembly/tree/master/pipeline/freebayes-polish), using 10x linked reads.

Assembly details are outlined in the Supplementary Note.

#### Genome annotation

RNA-seq reads from the six tissues were concatenated and used to assemble a transcriptome using Trinity (v.2.8.5)^66^. Repeat elements in the genome assembly were called using RepeatModeler (v.2.0) and masked using RepeatMasker (v.4.1.0)^67^. The genome was annotated using MAKER (v.2.31.10) and exonerate (v.2.2.0), with two rounds of SNAP training, following previous methods^68^. The *M.* *arenaria* transcriptome was used as input into the MAKER annotation, along with the proteins identified from five well-annotated bivalve genomes. Putative gene identification was made by BLASTP search of the uniprot database (accessed 2 March 2021) and the five well-annotated bivalve genomes using blast+ (v.2.10.0). Annotation details are outlined in the Supplementary Note.

Genome assembly statistics for the current and previous *M.* *arenaria* assemblies can be found in Supplementary Table 1. Genome size, GC content, scaffold N50 and contig N50 were calculated using BBTools stats.sh (v.38.86)^69^. Repeat content was estimated by running RepeatMasker (v.4.1.0) using the RepeatModeler repeat library generated above. BUSCO scores were calculated against the metazoa_odb10 database using BUSCO v.3 (ref. ^25^).

### MarBTN genome sequence analysis

#### Sample collection, DNA extraction and sequencing

MarBTN samples were collected from highly neoplastic clams from Maine and New York, USA, and PEI, Canada (Fig. 1a and Supplementary Table 2). Several MarBTN samples were previously reported (those collected between 2009 and 2014)^6,46^ and remaining samples (those collected between 2020 and 2022) were shipped live on ice from a seafood supplier in Maine. Hemolymph was drawn and screened for highly neoplastic animals (as above) and genomic DNA was extracted using the protocol previously used (DNeasy Blood and Tissue kit, QIAGEN)^6,46^. Two healthy clams were collected and DNA was extracted from the siphon or mantle tissue as reported previously^6^, in addition to the healthy reference clam. Previous reports of likely BTN in *M.* *arenaria* (Fig. 1a; denoted by x) are described in the Supplementary Note.

All samples were sequenced on an Illumina HiSeq (paired end 150-bp reads, Genewiz). Healthy tissue and cancer hemolymph were sequenced using a full lane with a target read depth of 50×. Paired tissue samples for a subset of cancer samples were sequenced with a target read depth of 30×. Illumina sequences were purged of optical duplicates using BBTools clumpify (v.38.86)^69^, trimmed using trimmomatic (v.0.36) with a read quality threshold of 20 and mapped to the reference genome using BWA-MEM^70^ with default settings.

#### SNV calling

SNVs and indels were called using somatypus (v.1.3), a platypus-based variant calling pipeline designed for closely related cancer data without a paired normal sample, ideal for transmissible cancer genomes^12^. Variants were called as present in a healthy clam if they were called by somatypus and supported by >3 reads. For cancer samples, we used more stringent thresholds to eliminate contaminating host DNA from being called as cancer alleles. Paired host tissue samples proved to be too highly contaminated by cancer to be useful and were only used as a downstream confirmation that we were eliminating host alleles with our read thresholds. Unlike mammalian transmissible cancers, which form solid tumors and allow collection of uncontaminated healthy host DNA, BTN disseminates into the tissues of the host as the cancer progresses, resulting in tissue samples that include significant BTN cells in late stages of the disease; however, we find DNA extracted from hemolymph of animals with late-stage disease to be so highly composed of BTN cells and so few host hemocytes (Extended Data Fig. 1) that we were able to effectively remove host variants using these thresholds. Thresholds for SNV calling are described in the Supplementary Note.

We used median allele frequency of MarBTN-specific homozygous nuclear SNVs in copy number 2 regions as a proxy for cancer isolate purity and host tissue purity, as shown in Extended Data Fig. 1.

#### LOH region identification

To call genome regions where one of the two original founder haplotypes was lost in one sub-lineage but retained by the other sub-lineage (termed LOH for loss of heterozygosity), we focused on SNVs for which we had high confidence that they came from the founder clam germline, using methods described in the Supplementary Note. A region with germline SNVs transitioning to homozygous from heterozygous (with the ancestral heterozygous state being captured in the other sub-lineage) would indicate regions that had lost a parental haplotype in the homozygous sub-lineage. We then calculated signature S mutation fraction and dN:dS ratio for each and plotted the values against the threshold used for the test calling (Extended Data Fig. 2c–e). To validate that our LOH calling method was successfully removing LOH regions we filtered for a different set of SNVs than those used to call LOH: sub-lineage-specific founder variants (variants found in a healthy clam and all individuals of one sub-lineage but none in the other sub-lineage). The density of USA-specific founder variants SNVs was 36× higher in PEI LOH regions versus non-LOH regions and PEI-specific founder variants SNVs was 20× higher in USA LOH regions versus non-LOH regions (Extended Data Fig. 2b), confirming these regions were likely lost from the other sub-lineage.

#### MarBTN phylogeny

To build the phylogeny in Fig. 1e we concatenated all variant loci into an alignment for all eight cancer samples with the reference genome sequence at those loci as the tree root. SNVs found in any healthy clam samples were excluded before this analysis, as nearly all those SNVs were likely present in the founder clam. SNVs in LOH regions were also excluded to remove founder variants from the sub-lineage branches. We then used R package ‘ape’ (v.5.5) to calculate the pairwise distance between sequences using the dist.dna(model = ‘raw’) function, build a neighbor-joining tree using the and nj() function and calculated bootstrap support using the boot.phylo() function, revealing high confidence (100 of 100) at all nodes.

#### Mutational signature extraction and fitting

**Extended Data Fig. 10:**
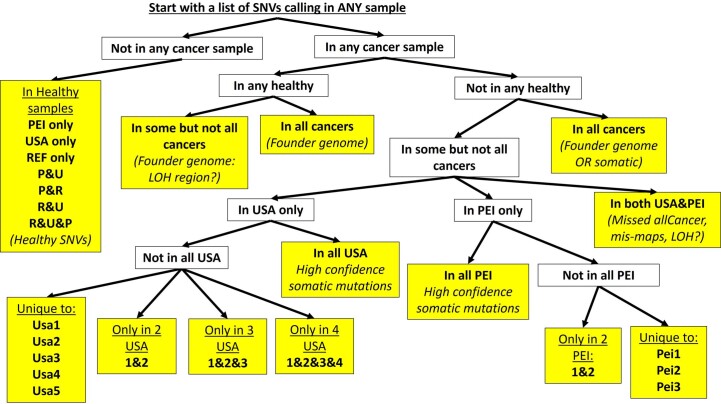
SNV binning strategy for analysis. Flowchart of our strategy to separate SNVs into bins for de novo signature extraction, based on which sample(s) each SNV was called in. Many of these bins were also used in other analyses, as indicated in the manuscript. The starting point refers to a vcf file of every SNV that was called in at least one of the eleven sample (three healthy, eight cancer) sequenced in this study. Bins highlighted in yellow indicate non-overlapping SNV bins used to for signature extraction.

We categorized SNVs into 25 bins based on which samples they were found in and the MarBTN phylogeny (see Extended Data Fig. 10 or code reference below). We further divided each SNV bin by annotated genome regions into additional nested bins (full genome, genes, exons, CDS, 5′ UTR and 3′ UTR), with the thought that some mutational processes may have different exposures across the genome. We used Helmsman (v.1.5.2)^71^ to count SNVs for each bin in their trinucleotide context and R package ‘Biostrings’ (v.2.54.0) to count trinucleotide opportunities in each genome region. We performed de novo signature extraction on this dataset using R package ‘sigfit’ (v.2.0.0)^72^, correcting for opportunities in each genome region. The unbiased estimate for the best number of signatures to fit our data was 3, though extracting four signatures revealed a signature of unmistakable resemblance to COSMIC signature 1 (CpG > TpG), so we proceeded with four signatures. SNV bins were then reanalyzed with these four signatures, again correcting for mutational opportunities, to reveal the fraction of SNVs in each category that could be attributed to each signature.

#### Cancer dating

To estimate the age of the MarBTN lineage we only wanted to consider likely somatic mutations, so we excluded regions that were called as LOH in either sub-lineage from these analyses (as true founder SNVs in a region lost in one sub-lineage would appear to be unique to the other sub-lineage and could be falsely considered to have occurred after the divergence of the sub-lineages if those regions were not removed). We only included genomic SNVs for this analysis, as there were a limited number of MarBTN-specific mitochondrial SNVs and they displayed a different mutational profile than genomic mutations. We then filtered remaining SNVs from each MarBTN sample to remove any SNVs that were found in a healthy clam or the other sub-lineage using the same thresholds as described above (SNV calling). We have high confidence that the remaining SNVs for each sample should be somatic mutations that occurred since the time the two sub-lineages diverged (as the MRCA). We counted the number of mutations in their trinucleotide contexts using Helmsman^71^ for each MarBTN sample and fitted this to our de novo extracted mutational signatures to estimate contributions of each of the four signatures. We then performed a linear regression of the mutation count attributed to each signature for each sample against the date the sample was collected (Extended Data Fig. 5). We performed regression across USA samples only, with the thought that this set would be less susceptible to small changes in mutation rates between the sub-lineages and would not be confounded by the timing or number of copy number differences between the sub-lineages. Within the USA sub-lineage, Sig5′ was the best fit with time. When considering PEI samples, SigS seemed to be more clock-like, in that PEI samples fall within the 95% CI of the USA regression. Additionally, to test whether structural mutation types that were higher in USA than PEI were due to sampling date, we performed the same analysis on somatic tandem duplications, somatic translocations, total Steamer insertion sites and total mitochondrial D-loop copies.

The *x* intercept of the regressions calculated above indicates the age of the MRCA of the two sub-lineages (when mutation count separating them equals zero). To estimate the total age of the cancer, we first estimated the number of somatic SigS mutations in the trunk of the MarBTN lineage (SNVs shared by all MarBTN samples) and we then used this estimation to further estimate the total number mutations as described in the Supplementary Note.

#### dN:dS

We ran R package ‘dNdScv’ (v.0.0.1.0)^38^ to calculate global dN:dS, the overall ratio across all genes in the genome, for each SNV subset in Fig. 2g (details are provided in the Supplementary Note). We also calculated dN:dS for individual genes. We filtered for genes under significantly positive or negative selection (corrected *P* value < 0.05). For the five hits generated when dN:dS was run for somatic mutations, we performed an NCBI blastp query for each of these genes. We checked each gene visually/manually using IGV, noting that in each case nearly all SNVs seem to be on a single haplotype. We calculated the dN:dS for SNVs found in any healthy clam for each of these five genes, removing one that was also under positive selection in the observed healthy clam genomes (presumed to be due to missed founder variants in a gene under positive selection in the healthy clam population). Results and notes for each gene are summarized in Supplementary Table 3.

#### Copy number calling

Most cancer copy number calling tools rely on having paired tissue samples; we instead developed a custom copy number calling script that uses cn.mops (v.1.32.0)^73^ to call read depth and depth relative to the reference clam (MELC-2E11) to determine copy number, with the assumption that this reference clam is diploid. The Supplementary Note provides the details and validation with allele frequency using bedtools (v.2.29.1)^74^.

To estimate duplication timing, we filtered for 100-kB segments that were called CN4 in both USA and PEI sub-lineages. We calculated VAFs for founder germline variants (found in all cancers and at least one healthy sample) and for high-confidence somatic mutations in each sub-lineage by taking the mean VAF for each SNV across the five USA samples and the three PEI samples. For each 100-kB segment, we calculated the fraction of 2/4 somatic mutations by taking mutations with VAF 0.375–0.625 and dividing by total mutations.

#### Structural variant and telomere calling

We used DELLY (v.0.8.5)^75^ to call deletions, small (<100 bp) insertions, tandem duplications, inversions and translocations in each sample individually from split read mapping. DELLY is sensitive to read depth, so we subsampled all sample sequences to only include 600,000,000 reads (which is a lower count than the lowest sequenced sample) before running DELLY using ‘samtools view -s’. We only considered SVs supported by reads mapping to precise breakpoints in the genome. We used default settings, except for setting a minimum paired end read mapping quality threshold to 30 to minimize false positives. We merged all called SVs into a single file based on shared breakpoints. We removed SVs called in the reference clam from all samples and compared the number of each SV type and size of each intra-chromosomal SV type. To narrow in on high-confidence somatic SVs we then filtered out SVs found in any healthy clam or the opposite sub-lineage from each sample (similar to our approach for identifying somatic SNVs) and compared the number and size of SVs. To compare SV counts between healthy/MarBTN and USA/PEI, we used a two-sided *t*-test (unequal variance) and to compare sizes we used a two-sided Wilcoxon signed-rank test.

We used telseq (v.0.0.2)^76^ using default settings to estimate telomere lengths. Telseq takes raw bam alignments for all samples (generated above) as an input and uses TTAGGG-repeat content to estimate mean telomere length for each sample as an output (Fig. 3e).

#### Identifying Steamer insertion sites

We called Steamer insertion sites in all samples via a custom pipeline which uses split reads that map to both the reference genome and Steamer itself (details are provided in the Supplementary Note).

We noticed a bias for ATG in positions 7–9 in both our upstream and downstream Steamer flanking reads. To investigate this bias, we extracted the 35 bp surrounding each Steamer insertion sites from the reference genome (15 bp upstream, 5 bp TSD and 15 bp downstream) using bedtools getfasta^74^. We then counted the number of occurrences of each nucleotide at each position, normalized by the GC content of the genome (35%) and created logo plots using ggseqlogo. This bias held whether we looked at Steamer sites across all samples, just cancer samples, sites shared by all cancer samples, sites unique to the USA sub-lineage and sites unique to the PEI sub-lineage. For sites found in any cancer sample, we also counted the number of sites that had an ATG in positions 7–9 upstream, downstream (note ATG in read in reverse is CAT) and both upstream and downstream. Compared to the frequency expected based on the frequency of ATG in the genome (2.2% of trinucleotides), these sites were 8.5, 7.4 and 44.6 times more frequent than expected by chance, respectively.

To investigate where Steamer inserted relative to genes, we found the closest gene to each insertion site using bedtools closest^74^, excluding insertion sites within genes. There was a noticeable bias in the 1–2 kB upstream genes (Extended Data Fig. 8) and these genes were more likely to be cancer-associated than expected by chance, as described in the Supplementary Note.

#### TE copy number analysis

We did not observe Steamer in our RepeatModeler run on the reference genome, likely due to it being present at low copy number in healthy clams and thus not clearing the threshold to be called as a repeat element. To capture other repeat elements such as Steamer that might have a copy number in MarBTN but be low in the reference genome, we ran REPdenovo^77^, a repeat element identifier that can be run on raw WGS data, as opposed to the assembled genome required for RepeatModeler. We ran REPdenovo on the healthy reference clam (MELC-2E11), a USA MarBTN sample (MELC-A11) and a PEI MarBTN sample (PEI-DN08) to capture repeat elements at high copy number in either sub-lineage, as well as a healthy clam to control for biasing repeat element identification toward MarBTN. We then ran RepeatClassifier, a component of RepeatModeler used for classifying repeats based on sequences, on the output repeat elements.

To generate a consensus repeat library, we used CD-HIT (v.4.8.1)^78^ to merge the libraries generated from the RepeatModeler and REPdenovo runs, using the same CD-HIT settings as those used by RepeatModeler itself to merge repeats with greater than 80% identity (-aS 0.8 -c 0.8 -g 1 -G 0 -A 80 -M 10,000). We then used BWA-MEM to map reads from each sample to the repeat library and calculated the average read depth across each repeat element and normalized by read depth across the genome, calculated previously, to yield an estimate of the number of copies of each repeat element in each sample. Note that this copy number is relative to the haploid genome for all samples.

For each repeat element, we calculated the average copy number among our three healthy clams, eight MarBTN samples and each MarBTN sub-lineage individually. We calculated the ratio of copies in healthy clams versus MarBTN samples and PEI sub-lineage versus the USA sub-lineage, followed by a two-tailed unequal variance *t*-test to calculate the significance of each difference (Fig. 4d). We removed repeats with fewer than one copy in any sample, as these likely represent TEs that are only present in a subset of the clam population and would yield a highly significant difference simply due to the absence in some samples and presence in others. We additionally divided and plotted the dataset by repeat type classified by RepeatClassifier (DNA transposon, LTR, LINE, rolling circle, rRNA, simple repeat, SINE, snRNA or tRNA). We performed chi-squared tests to determine whether certain elements were higher copy number in one group versus another. The magnitude of repeat expansions may be overestimated as we are comparing an average from three difference clams to an average from eight samples of a clonal lineage; however, the strong skew toward more copies in MarBTN compared to healthy clams indicates that either (1) the founder clam had more copies of many TEs than the healthy animals sequenced here or (2) many TEs have increased their copy number through somatic expansion.

#### Mitochondrial analysis

We mapped each whole-genome sequenced sample to the previously published mitochondrial genome^45^ using BWA-MEM^70^. We then ran somatypus^12^ using default settings to call SNVs and indels. We excluded SNVs around the multi-copy region in positions 12,060–12,971. We did not see evidence of heteroplasmy outside this region, so an SNV was counted as present if it was present in a sample at >0.5 VAF. To infer relatedness of mitochondrial genotypes we built a neighbor-joining tree, as conducted for genome SNVs, from an alignment of sequences built by concatenating all variant allele positions versus the reference mitochondrial genome (170 loci).

To look at mutational biases, we included 12 possible single-nucleotide substitution types rather than the traditional 6, as the heavy/light strand differences of mtDNA result in unequal C/G and A/T in the forward or reverse direction (forward: A, 0.29%; T, 0.37%; C, 0.12%; and G, 0.23%). We counted SNVs of each substitution type for SNVs found in healthy clams (39), shared among all MarBTN samples but not found in healthy clams (13), those found in all samples of the USA (21) or PEI (26) sub-lineages and all high-confidence somatic mutations (50; those found in only a subset of MarBTN samples). We also calculated the expected number of substitutions of each type based on the nucleotide content of the mitochondrial genome assuming no mutational biases for comparison.

We used dndscv^38^ as described previously to calculate the global dN:dS in the mitochondrial genome. We calculated dN:dS for SNVs found in healthy clams, SNVs shared among all cancer samples but not found in healthy clams and high-confidence somatic mutations (those found in just the USA or PEI sub-lineages). 95% CIs from dndscv are quite large due to the small number of coding mitochondrial mutations in our samples used for this calculation.

We calculated the read depth at each position using SAMtools depth. To estimate the number of copies of the D-loop region, we calculated the average read depth in positions 12,300–12,500 relative to the average read depth across the full mitochondrial genome, excluding that region. This region was chosen because it is within the multi-copy D-loop region but should not have reads that border the duplication breakpoint or the insertion that is only present in some copies and may cause errors in amplification due to its G-rich sequence. Copy numbers were compared between the groups using a *t*-test (two-sided and unequal variance).

We confirmed the presence of a D-loop tandem duplication in a healthy clam using inverse PCR (Extended Data Fig. 7e), with outward-facing primers that would only amplify if the copies or the region are in tandem (Supplementary Table 4). PCR amplification and long-read assembly confirms tandem duplication of the region (details provided in the Supplementary Note).

#### RNA-sequence analysis

Samples from multiple tissues were collected and RNA was extracted/sequenced as described above for two healthy clams (to add to the previously RNA-sequenced reference clam, MELC-2E11) and for hemocytes only for two additional healthy clams. Hemolymph was drawn from five heavily diseased clams and MarBTN isolates were further purified by allowing to settle for 1 h in a 24-well plate at 4 °C. Remaining host hemocytes adhered to the plate and purified MarBTN cells were gently collected by pipetting. RNA was extracted and sequenced as described above (six samples per Illumina HiSeq 4000 lane for 20–30 million reads per sample).

We aligned reads for all samples to the indexed annotated genome using STAR (2.7.5a_2020-06-29)^79^ and quantified reads mapped per gene using quantMode GeneCounts. We confirmed that MarBTN isolates were all part of the USA sub-lineage at 48 of 48 mitochondrial loci differentiating USA versus PEI and that the VAFs of USA-specific mitochondrial SNVs were 96–99% in all samples, confirming high BTN purity. We merged counts per gene for all samples and ran DESeq2 (v.1.26.0)^80^, using tissue (or BTN) as the condition on which to test differential expression. We performed principal-component analysis by applying variance-stabilizing transformation using vst() and plotPCA() from the DESeq2 package. We determined the top tissue-specific genes for each tissue by comparing each to the five others using DESeq2, sorting by the ‘stat’ output and taking the top 100 overexpressed genes for each tissue. We normalized read counts for each sample by calculating total mapped reads and multiplying so that each sample totaled the same number of reads as the maximum sample. We then performed hierarchical clustering on expression of the 600 tissue-specific genes using the pheatmap package with clustering_distance_cols = ‘canberra’. For individual gene comparisons of MarBTN versus healthy samples, we compared MarBTN separately to hemocytes and to non-hemocyte solid tissues. Bar plots are comparisons of normalized read counts per gene, whereas statistical results for differential expression are adjusted *P* values from DESeq2.

#### Statistics and reproducibility

No statistical method was used to predetermine sample size, but our sample sizes are similar to those reported in previous publications^11,14^. Two cancer samples were excluded from this analysis due to high host contamination of samples, as described in Extended Data Fig. 1. The experiments were not randomized. The investigators were not blinded to allocation during experiments and outcome assessment. For all *t*-tests, data distribution was assumed to be normal but this was not formally tested.

### Reporting summary

Further information on research design is available in the Nature Portfolio Reporting Summary linked to this article.

### Supplementary information

Supplementary Information Supplementary note.

Reporting Summary

Supplementary Table 1 Supplementary Tables 1–6.

### Source data

Source Data (all figures) Source data for all figures and extended data figures.

## Data Availability

Raw sequence data and the assembled genome are available via NCBI BioProject PRJNA874712 (https://www.ncbi.nlm.nih.gov/bioproject/874712). This study also used the GenBank (KF319019.1, NC_024738.1, GCA_011752425.2, GCF_002022765.2, GCF_002113885.1, GCF_902652985.1 and GCF_902806645.1) and Uniprot (release 2021_01) databases. Data outputs can be obtained by running the supplied code on the raw data or on request. Source data for all figures and extended data figures are available in the source data file. All other data supporting the findings of this study are available from the corresponding author on reasonable request. Source data are provided with this paper.

## Code availability

All code is available on GitHub (https://github.com/sfhart33/MarBTNgenome), including all dependencies with version numbers. The Supplementary Note contains individual commands for genome assembly (triangular bullets) and scripts corresponding to each written genome analysis method section (bullets). Analysis was performed with an on-premises Linux server running Ubuntu v.16.04. The Linux server was equipped with four Intel Xeon Gold 6148 CPUs and 250 GiB system memory. Note that code was written for our institute’s working environment and thus some scripts may need to be altered manually to reproduce this analysis.
