# Supplementary material for: Centuries of genome instability and evolution in soft-shell clam, Mya arenaria, bivalve transmissible neoplasia
Source: Nat Cancer. 2023 Oct 2;4(11):1561–74. doi: 10.1038/s43018-023-00643-7 (PMC10663159; doi:10.1038/s43018-023-00643-7)
Supplement: Supplementary file 1 — Supplementary note. [file 43018_2023_643_MOESM1_ESM.pdf]

nature cancer

Article https://doi.org/10.1038/s43018-023-00643-7

Centuries of genome instability and evolution in soft-shell clam, *Mya arenaria*, bivalve transmissible neoplasia

In the format provided by the authors and unedited

Mya arenaria genome assembly (extended methods and commands)

HMW DNA extraction for PacBio sequencing

High molecular weight (HMW) DNA, used for PacBio sequencing, was extracted from ~50 mg snap-frozen mantle tissue using a modified CTAB extraction protocol. CTAB isolation buffer (2% CTAB, 1.4 M NaCl, 20 mM EDTA, 100 mM Tris-HCl, pH 8.0) was preheated to 60°C in a water bath. Tissue was minced, then ground with a pestle in 500 µL 60°C CTAB isolation buffer in a 1.7 ml microcentrifuge tube. 20 µL proteinase K was added and the sample was incubated at 60°C for 10 h on a shaker (200 rpm), then held at room temperature. Sample was extracted once with the addition of 500 µL chloroform-isoamyl alcohol (24:1), mixing gently but thoroughly. This produces two phases, an upper aqueous phase which contains the DNA, and a lower chloroform phase that contains some degraded proteins, lipids, and many secondary compounds. The sample was spun at 6,000 × g for 10 min at room temperature to concentrate phases. Aqueous phase was removed with a wide bore pipet, transferred to a new microcentrifuge tube. 2/3 volumes cold isopropanol (237 µL) was added and inverted gently to precipitate nucleic acids. HMW DNA was spooled out with a glass hook and transferred to a 2 mL microcentrifuge tube containing 1 mL wash buffer (76% ethanol, 10 mM ammonium acetate) for 20 minutes. HMW DNA was spun down (6,000 × g for 10 min) after a minimum of 20 min of washing. Supernatant was poured off carefully and allowed to air dry briefly at room temperature. HMW DNA was resuspended in 200 µL TE (10 mM Tris-HCl, 1 mM EDTA, pH 8.0). RNase A (DNase-free, 10 mg/mL, Thermo Scientific, Waltham, MA) was added to a final concentration of 25 ug/ml (0.5µL) and incubated 30 min at 37°C. Sample was diluted to 2 volumes with TE, 10 M ammonium acetate was added to a final concentration of 2.5 M, sample was mixed, 1.2 mL 100% ethanol was added, and sample was gently inverted to precipitate HMW DNA. HMW was spun down (10,000 × g for 10 min at 4°C). Sample was air dried and resuspended in 200 µL TE buffer overnight at 4°C.

10X Chromium sequencing

High molecular weight genomic DNA was isolated from reference animal (MELC-2E11) tissue using the MagAttract HMW DNA Kit (Qiagen), quantified using Qubit 2.0 (Life Technologies) and fragment size determined using the Agilent 2200 TapeStation. Average fragment size exceeded 50 Kb. Approximately 1 ng of DNA was loaded on the Chromium Genome Chip (10X Genomics). Whole genome sequencing libraries were prepared using Chromium Genome Library & Gel Bead Kit v.2, Chromium i7 Multiplex Kit and Chromium Controller according to 10X Genomics instructions. The resulting library was indexed and sequenced on 0.75 lanes of a single flow cell on the Illumina HiSeq X Ten system, generating 150-bp paired-end reads.

Unsuccessful assembly using 10X Chromium data

An attempt to assemble a genome of the MELC-2E11 reference animal using 10X sequencing was unsuccessful at creating a highly contiguous genome, likely due to the higher amount of repeats in the bivalve genome than in the human genome. However, we report it here for transparency of our assembly attempts. *De novo* assembly was performed using Supernova (v2.1.1). Assembly was conducted with the command:

- supernova run --id=10X_MELC-2E11-100 --fastqs=/home/mmetzger/10XChromiumSized/Data --description="MELC-2E11 sized 10X assembly " --maxreads=all --accept-extreme-coverage

Secondary attempts at assembly were conducted using down-sampled subsets of the data (75%, 666928259 reads; 50%, 444618839 reads, and 25%, 222309414 reads), using the read numbers listed above for the option “--maxreads". The pseudohap2 output was used (Mar.3.1.1 Myaare100B_pseudohap2.1.fasta).

FALCON-Unzip diploid assembly commands

- bam2fasta -o Marenaria.3.2 *.subreads.bam
- fc_run fc_run_marenaria.cfg &> run1.log &
- mv all.log all0.log

➢ fc_unzip.py fc_unzip_marenaria.cfg &> run1.std &

Several modifications were made to default configuration parameters, including changing to "pwatcher_type=blocking" and lowering the memory per job in fc_unzip_marenaria.cfg. Configuration files are found on github (fc_run_marenaria.cfg and fc_unzip_marenaria.cfg). FALCON-Unzip version was pbbioconda-0.0.5 and was used with python 3.7. This FALCON-Unzip pipeline resulted in a primary contig assembly (Mar.3.2.2_cns_p_ctg.fasta) and an alternate haplotig assembly (Mar.3.2.2_cns_h_ctg.fasta).

Scaffolding with Hi-C using FALCON-Phase

Chromatin conformation capture data was generated using a Phase Genomics (Seattle, WA) Proximo Hi-C Animal Kit, which is a commercially available version of the Hi-C protocol. Following the manufacturer's instructions, intact cells from two adductor muscle samples from the same reference clam (MELC-2E11) were crosslinked using a formaldehyde solution, digested using the Sau3AI restriction enzyme, and proximity ligated with biotinylated nucleotides to create chimeric molecules composed of fragments from different regions of the genome that were physically proximal *in vivo*, but not necessarily genomically proximal. Molecules were pulled down with streptavidin beads and processed into an Illumina-compatible sequencing library. Sequencing was performed on an Illumina NextSeq 500, generating a total of 313,340,002 PE150 read pairs.

The Hi-C reads, primary contigs, and alternate haplotigs (Mar.3.2.3_curated.haplotigs.FALC.fasta) were provided as input to FALCON-Phase (https://phasegenomics.github.io/2019/09/19/hic-alignment-and-qc.html) to correct likely phase switching errors. All other options were set to default, except for the options which specify restriction enzyme motifs in the library (GATC) and the number of iterations to perform (100,000,000). Phased contigs were output in pseudohap format, creating one complete set of contigs for each of the two phased assemblies from the diploid genome of the reference individual (arbitrarily named Phase 0 and Phase 1).

Reads were aligned to the resulting Phase 0 contig assembly 3etwork_mussel.phased.0.fasta following Phase Genomics' standard Hi-C alignment protocol. Briefly, reads were aligned using BWA-

MEM with the -5SP and -t 8 options specified, and all other options default. SAMBLASTER was used to flag PCR duplicates, which were later excluded from analysis. Alignments were then filtered with samtools using the -F 2304 filtering flag to remove non-primary and secondary alignments. These alignments, along with the primary contigs and alternate haplotigs were used as inputs to the scaffolding process.

Phase Genomics' Proximo Hi-C genome scaffolding platform was used to create chromosome-scale scaffolds from the Phase 0 assembly, following the same single-phase scaffolding procedure described in Bickhart et al.. As in the LACHESIS method, this process computes a contact frequency matrix from the aligned Hi-C read pairs, normalized by the number of restriction sites (GATC) on each contig, and constructs scaffolds in such a way as to optimize expected contact frequency and other statistical patterns in Hi-C data. Approximately 120,000 separate Proximo runs were performed to optimize chromosome assignment and scaffold construction in order to make the scaffolds as concordant with the observed Hi-C data as possible. This process resulted in a set of 17 chromosome-scale scaffolds containing 1,212 Mbp of sequence (99.89% of the phase 0 assembly) with a scaffold N50 of 78.4 Mbp. Juicebox was used to correct likely scaffolding errors, though no breaks for mis-joined contigs were introduced at this stage in order to maintain exact contig relationships with the Phase 1 assembly.

Separately, Hi-C data were aligned to a concatenated Phase 0 and Phase 1 assembly using the standard protocol cited above. Because this would cause Hi-C data for most homozygous regions to have a MAPQ of 0 (among possible other issues), this alignment emphasizes phase-specific Hi-C relationships. These alignments and the Phase 0 scaffolds were passed to FALCON-Phase's bamfilt (-f 20 -m 10), bam2 binmat (default options), and phase (-n 100000000 -s 10) steps to generate new phasing metadata intended to correct latent phasing issues not detected during the earlier contig phasing step.

Juicebox was again used to correct remaining scaffolding errors in Phase 0, including introducing a single break into each of eight suspected mis-joined contigs and two breaks into one suspected double-misjoined contig, based on the appearance of Hi-C signals consistent with chimeric joins. These scaffolding changes were replicated to Phase 1, and new scaffolds for each phase were generated using the juicebox_assembly_converter.py script (https://github.com/phasegenomics/juicebox_scripts). In these final

scaffolds, both Phase 0 and Phase 1 included 17 scaffolds spanning 99.7% (1,204 Mbp in Phase 0 and 1,214 Mbp in Phase 1) of input with a scaffold N50 of 70.2 Mbp in Phase 0 and 71.4 Mbp in Phase 1 (Mar.3.3.2_p0_PGA_assembly.fasta and Mar.3.3.2_p1_PGA_assembly.fasta). The 17 scaffolds from both of these two haploid assemblies were compiled into Mar.3.3.2_p0p1_PGA_assembly_17.fasta using a custom perl script two_fasta_prefix_compile_firstX.pl

- perl two_fasta_prefix_compile_firstX.pl Mar.3.3.2_p0_PGA_assembly.fasta Mar.3.3.2_p1_PGA_assembly.fasta p0_ p1_ Mar.3.3.2_p0p1_PGA_assembly_17.fasta 17

Genome Gap-Filling and Polishing commands

For gap-filling with PBJelly, each PacBio fastq file was split into 4 subfiles, using the custom perl script splitfastqXfiles.pl. Only captured gaps were filled (no inter-scaffold gaps) using the option "—capturedOnly" during the "support" step.

- Jelly.py setup Protocol_MELC.xml
- Jelly.py mapping Protocol_MELC.xml
- Jelly.py support Protocol_MELC.xml -x "—capturedOnly"
- Jelly.py extraction Protocol_MELC.xml
- Jelly.py assembly Protocol_MELC.xml -x "—nproc=20"
- Jelly.py output Protocol_MELC.xml

The output of PBJelly (Mar.3.3.3_jelly.out.fasta) renamed all scaffolds to Contig0-Contig33, so names were corrected manually based on PBJelly liftOverTable.json (Mar.3.3.3_jelly.out_name.fasta), and the two haploid genomes were separated (using commands listed in PBJellyRenaming.txt) to generate the haploid gap-filled assemblies (Mar.3.3.3.p0.fasta and Mar.3.3.3.p1.fasta)

Direct use of short reads to polish a highly heterozygous genome is likely to introduce more errors than it corrects, due to the mapping of reads from both haplotypes to a single haploid genome. Therefore, we used a phase-aware polishing strategy, using the 10X linked reads generated above, modified from the pipeline described in the vertebrate genome project (https://github.com/VGP/vgp-

assembly/tree/master/pipeline/freebayes-polish). Both phases (p0 and p1) of the scaffolded, and gap-filled diploid genome were concatenated into a single diploid reference file with 34 scaffolds, and the linked-read-aware mapper Longranger (v2.2.2) was used to map the 10X reads to the diploid gap-filled assembly (Mar.3.3.3_jelly.out_name.fasta), and the output was indexed using samtools (v1.9). FreeBayes (v1.3.1) and Bcftools (v1.10.2) were used to call SNPs in reads that mapped uniquely to one location on one haplotype, under stringent conditions, using a Q>30 filtering of both the Longranger mapping calls and FreeBayes variant calls. For Bcftools, filters allowed only homozygous ALT alleles (GT="A"), as the reference assembly used was a concatenated diploid assembly instead of a haploid one. 1,862,877 variants were called. The resulting polished, concatenated diploid assembly (Mar.3.4.6.p0p1_Q30Q30A.fasta) was split into the two polished haploid genomes and renamed (Mar.3.4.6.p0_Q30Q30A.fasta and Mar.3.4.6.p1_Q30Q30A.fasta). Commands for running of polishing and renaming of the assembly are available (LongrangerFreeBayesBcftoolsPolishing.txt).

The Phase 0 and Phase 1 assembled, scaffolded, and polished haploid genomes represent the two genomes found in the diploid reference individual. One must be chosen to be the reference for mapping and analysis of other genomes, so Phase 1 was selected as the primary reference genome for annotation and further use, as it contained the first endogenous *Steamer* insertion site that was initially reported. This site is polymorphic in *Mya arenaria* populations and was not present in Phase 0.

RNA extraction

In order to create a transcriptome assembly that would include transcripts expressed in different tissue types across the clam, seven tissues from the reference animal (MELC-2E11) frozen at -80°C in RNAlater (Invitrogen, Waltham, MA) were used for RNA extraction (1, mantle; 2, foot; 3, siphon; 4, stomach; 5, adductor muscle; 6, gills; and 7, hemocytes). Solid tissues were homogenized with a disposable plastic mortar and pestle in liquid nitrogen before extraction with the Qiagen RNeasy kit (Qiagen, Hilden, Germany), eluting in 60 µL elution buffer. DNase I (2 µL, 2,000 U/ml, RNase-free, New England Biolabs, Ipswich, MA), 10× DNase buffer, and water was then added to a total of 100 µL, and the reaction was

incubated for 1 h at room temperature. Then 250 µL ethanol was added and mixed by pipette, and it was added to a second Qiagen RNeasy column. The RNeasy protocol was followed, skipping the RW1 step, adding 500 µL RPE 2×, and eluting in 40 µL elution buffer. RNA samples (excluding the stomach due to possible contamination with RNA from clam food) were then sequenced on a single Illumina HiSeq 4000 lane for 20-30 million reads per sample (Genewiz, Leipzig, Germany).

RNAseq reads from the six tissues were concatenated to create single files for each read direction and used to assemble a transcriptome using Trinity (v2.8.5):

- Trinity --seqType fq --max_memory 200G --CPU 16 --trimmomatic --full_cleanup --left MELC-2E11_R1_allfiles-cat.fastq.gz --right MELC-2E11_R2_allfiles-cat.fastq.gz

Genome annotation commands

Repeat elements in the genome assembly were called using RepeatModeler (v2.0), and repeat elements were masked using RepeatMasker (v4.1.0):

- RepeatModeler -database Mar.3.4.6.p1_Q30Q30A -pa 20 -LTRStruct
- RepeatMasker -pa 20 -lib $i-families.fa Mar.3.4.6.p1_Q30Q30A.fasta

The five well-annotated bivalve genomes used for gene annotation were *Mytilus coruscus*, GCA_011752425.2; *Crassostrea virginica*, GCF_002022765.2; *Mizuhopecten yessoensis*, GCF_002113885.1; *Pecten maximus*, GCF_902652985.1; and *Crassostrea gigas*, GCF_902806645.1, concatenated into a single file (CgiCviMcoPmaMye_protein.fasta). Putative gene identification was made by BLASTP search of the uniprot database (accessed 2021-03-02) and the proteins identified from the five well-annotated bivalve genomes using blast+ (v2.10.0). The top hit (with and e value <1e-6) was used for gene identification. Genes were labeled based on the most similar uniprot hit (if applicable with an e value <1e-6) and "-like" suffix or labeled as uncharacterized if only matching an uncharacterized bivalve gene or no gene at all. To account for multiple genes with the same uniprot hit, an additional numeric suffix was added to indicate additional hits to the same uniprot gene or uncharacterized genes (e.g. "TEN1-like_3", "uncharacterized_1199"):

- wget ftp://ftp.uniprot.org/pub/databases/uniprot/current_release/knowledgebase/complete/uniprot_sprot.fasta.gz
- gunzip uniprot_sprot.fasta.gz
- makeblastdb -in uniprot_sprot.fasta -out uniprot_sprot -dbtype prot
- blastp -query /home/metzgerm/MAKER_Mya/2020-09-11-Mar.3.4.6.p1-MAKER/snap02/2020-09-11_Mar_genome_snap02.all.maker.proteins.fasta -db uniprot_sprot -evalue 1e-6 -max_hsps 1 -max_target_seqs 1 -outfmt 6 -out 2020-09-11_Mar_genome_snap02.all.maker.proteins.fasta.blastp -num_threads 20
- makeblastdb -in CgiCviMcoPmaMye_protein.fasta -out CgiCviMcoPmaMye_protein -dbtype prot
- blastp -query /home/metzgerm/MAKER_Mya/2020-09-11-Mar.3.4.6.p1-MAKER/snap02/2020-09-11_Mar_genome_snap02.all.maker.proteins.fasta -db CgiCviMcoPmaMye_protein -evalue 1e-6 -max_hsps 1 -max_target_seqs 1 -outfmt 6 -out 2020-09-11_Mar_genome_snap02.all.maker.proteins.fasta.CgiCviMcoPmaMye_blastp -num_threads 20

MarBTN genome sequence analysis (extended methods and github)

All code is available on GitHub (https://github.com/sfhart33/MarBTNgenome), including all dependencies with version numbers. Bullets below indicate scripts corresponding to each written genome analysis method section.

Sample collection, DNA extraction and sequencing

Previous reports of likely BTN in *M. arenaria* (**Figure 1a**, x's) were collected from reports in which "disseminated neoplasia" or "hemic neoplasia" were diagnosed in *Mya arenaria* from the following publications:

1. Farley, C. A., Plutschak, D. L. & Scott, R. F. Epizootiology and distribution of transmissible sarcoma in Maryland softshell clams, Mya arenaria, 1984-1988. *Environ. Health Perspect.* **90**, 35–41 (1991).

2. Muttray, A. *et al.* Haemocytic leukemia in Prince Edward Island (PEI) soft shell clam (Mya arenaria): Spatial distribution in agriculturally impacted estuaries. *Science of The Total Environment* **424**, 130–142 (2012).

3. Reno, P. W., House, M. & Illingworth, A. Flow cytometric and chromosome analysis of softshell clams, Mya arenaria, with disseminated neoplasia. *Journal of Invertebrate Pathology* **64**, 163–172 (1994).

4. Sunila, I. Respiration of sarcoma cells from the soft-shell clam Mya arenaria L. under various conditions. *Journal of Experimental Marine Biology and Ecology* **150**, 19–29 (1991).

5. Cooper, K. R., Brown, R. S. & Chang, P. W. The course and mortality of a hematopoietic neoplasm in the soft-shell clam, Mya arenaria. *Journal of Invertebrate Pathology* **39**, 149–157 (1982).

6. AboElkhair, M. *et al.* Reverse transcriptase activity in tissues of the soft shell clam Mya arenaria affected with haemic neoplasia. *Journal of Invertebrate Pathology* **102**, 133–140 (2009).

7. AboElkhair, M. *et al.* Lack of detection of a putative retrovirus associated with haemic neoplasia in the soft shell clam Mya arenaria. *Journal of Invertebrate Pathology* **109**, 97–104 (2012).

8. Brousseau, D. J. & Baglivo, J. A. Field and laboratory comparisons of mortality in normal and neoplastic Mya arenaria. *Journal of Invertebrate Pathology* **57**, 59–65 (1991).

9. Delaporte, M. *et al.* Assessment of haemic neoplasia in different soft shell clam Mya arenaria populations from eastern Canada by flow cytometry. *Journal of Invertebrate Pathology* **98**, 190–197 (2008).

10. Leavitt, D. F. *et al.* Hematopoietic neoplasia inMya arenaria: Prevalence and indices of physiological condition. *Mar. Biol.* **105**, 313–321 (1990).

11. Le Grand, F. *et al.* Disseminated Neoplasia in the Soft-Shell Clam Mya arenaria: Membrane Lipid Composition and Functional Parameters of Circulating Cells. *Lipids* **49**, 807–818 (2014).

12. Lesser, M. P., Thompson, M. M. & Walker, C. W. Effects of Thermal Stress and Ocean Acidification on the Expression of the Retrotransposon Steamer in the Softshell Mya arenaria. *shre* **38**, 535–541 (2019).

13. Mateo, D. R., MacCallum, G. S. & Davidson, J. Field and laboratory transmission studies of haemic neoplasia in the soft-shell clam, Mya arenaria, from Atlantic Canada. *Journal of Fish Diseases* **39**, 913–927 (2016).

14. McLaughlin, S. M., Farley, C. A. & Hetrick, F. M. Transmission studies of sarcoma in the soft-shell clam, Mya arenaria. *In Vivo* **6**, 367–370 (1992).

15. Oprandy, J. J. & Chang, P. W. 5-Bromodeoxyuridine induction of hematopoietic neoplasia and retrovirus activation in the soft-shell clam, Mya arenaria. *Journal of Invertebrate Pathology* **42**, 196–206 (1983).

16. Reinisch, C. L., Charles, A. M. & Troutner, J. Unique antigens on neoplastic cells of the soft shell clam Myaarenaria. *Developmental & Comparative Immunology* **7**, 33–39 (1983).

17. Siah, A., McKenna, P., Berthe, F. C. J., Afonso, L. O. B. & Danger, J.-M. Transcriptome analysis of neoplastic hemocytes in soft-shell clams Mya arenaria: Focus on cell cycle molecular mechanism. *Results in Immunology* **3**, 95–103 (2013).

18. Siah, A., McKenna, P., Danger, J.-M., Johnson, G. R. & Berthe, F. C. J. Induction of transposase and polyprotein RNA levels in disseminated neoplastic hemocytes of soft-shell clams: Mya arenaria. *Developmental & Comparative Immunology* **35**, 151–154 (2011).

19. Siah, A., Delaporte, M., Pariseau, J., McKenna, P. & Berthe, F. C. J. Patterns of p53, p73 and mortalin gene expression associated with haemocyte polyploidy in the soft-shell clam, Mya arenaria. *Journal of Invertebrate Pathology* **98**, 148–152 (2008).

20. Siah, A., McKenna, P., Danger, J. M., Johnson, G. & Berthe, F. C. J. Expression of RAS-like family members, c-jun and c-myc mRNA levels in neoplastic hemocytes of soft-shell clams Mya arenaria using microsphere-based 8-plex branched DNA assay. *Results in Immunology* **2**, 83–87 (2012).

21. Sunila, I. & Farley, C. Environmental limits for survival of sarcoma cells from the soft-shell clam Mya arenaria. *Dis. Aquat. Org.* **7**, 111–115 (1989).

22. Taraska, N. G. & Anne Böttger, S. Selective initiation and transmission of disseminated neoplasia in the soft shell clam Mya arenaria dependent on natural disease prevalence and animal size. *Journal of Invertebrate Pathology* **112**, 94–101 (2013).

23. Walker, C. *et al.* Mass Culture and Characterization of Tumor Cells From a Naturally Occurring Invertebrate Cancer Model: Applications for Human and Animal Disease and Environmental Health. *The Biological Bulletin* **216**, 23–39 (2009).

24. Weinberg, J. R., Leavitt, D. F., Lancaster, B. A. & Capuzzo, J. M. Experimental Field Studies withMya arenaria(Bivalvia) on the Induction and Effect of Hematopoietic Neoplasia. *Journal of Invertebrate Pathology* **69**, 183–194 (1997).

- 02_Illumina_data_processing/02_map_to_genome.sh
- 02_Illumina_data_processing/00_sampling_map.R
- 02_Illumina_data_processing/01a_dedupe_and_trim.sh
- 02_Illumina_data_processing/01b_dedupe_and_trim_newsamples.sh

SNV calling

Sequencing resulted in a range of average sequencing depths of 57-90 at called SNV loci across samples. We normalized SNV calling thresholds to the average read depth for each individual sample, to avoid biasing calls in favor of more deeply sequenced samples. A variant was called as present in cancer

if it was present in at least one cancer sample at a depth of greater than 1/6 the average read depth for that sample (9-15 reads). Given a variant passed that criteria for at least one cancer sample, it was called in any other cancer sample if it was present at a depth greater than 1/16 the average read depth for that sample (3-5 reads). These thresholds were chosen to minimize the calling of host alleles or mis-mapped reads as cancer alleles, while also preventing the exclusion of real cancer alleles from our variant set.

We used median allele frequency MarBTN-specific homozygous nuclear SNVs in copy number 2 regions as a proxy for cancer isolate purity and host tissue purity, as shown in **Extended Data Fig. 1**. For non-reference healthy clams (the reference clam has no homozygous SNVs since one of the haplotypes is the reference genome), median allele frequencies were calculated from homozygous SNVs present in all samples. For cancer samples, median allele frequencies were slightly lower, attributed to the presence of host clam DNA, but remained >96%. Two MarBTN isolates that were excluded from this study due to high host DNA contamination were included on this analysis as contaminated sample controls. For samples for which paired tissue sequencing existed (3 of 8 cancer samples), we used the median allele frequencies of the same set of SNVs to estimate contamination of tissue by cancer DNA. Some samples contain a high amount of cancer DNA, making genome-wide differentiation between host and cancer SNVs difficult in tissue and leading us not to include paired tissue DNA in our analyses.

- 02_Illumina_data_processing/03_rename_for_somatypus.sh
- 02_Illumina_data_processing/04_run_somatypus.sh
- 03_SNV_analysis/02_initial_SNV_counts.R
- 06_Mito_analysis/05a_sample_purity_nuclear_and_mito.sh
- 06_Mito_analysis/05a_sample_purity_nuclear_and_mito.R

LOH region identification

To call genome regions where one of the two original founder haplotypes was lost in one sub-lineage but retained by the other sub-lineage (termed LOH for loss of heterozygosity), we focused on SNVs for which we had high confidence that they came from the founder clam germline–those found in all cancer

samples and at least one healthy clam. We calculated the allele frequencies for each of these SNVs in each cancer sample and flagged SNVs that were likely homozygous (above 0.8 frequency) for all samples in one sub-lineage, while heterozygous (less than 0.8) for all samples in the other sub-lineage. We included three representative samples from the USA sub-lineage in these calls (FFM-19G1, FFM-22F10 and NYTC-C9) so that calls were not biased by there being more USA samples than PEI. A region with SNVs transitioning to homozygous from heterozygous (with the ancestral heterozygous state being captured in the other sub-lineage) would indicate regions that had lost a parental haplotype in the homozygous sub-lineage. We looked at sliding windows of 50 heterozygous founder SNVs across each scaffold (independently for the PEI and USA sub-lineages) and counted the number of SNVs that were heterozygous -> homozygous discordant in the other sub-lineage. We found that windows with 10 or more discordant SNVs were the most effective for calling LOH regions (see below for validation of this threshold). We merged overlapping windows, for a total of 1,098 LOH windows for PEI (155 Mb, 12.8% of the genome) and 817 LOH windows for USA (98 Mb, 8.1% of the genome).

The amount of the genome that was called as in an LOH region was highly sensitive to the threshold of heterozygous -> homozygous discordant SNVs used to call LOH windows. We used two metrics to determine the best threshold, signature S mutation fraction and dN/dS. Both metrics are proxies for somatic mutations, with lower values corresponding to more founder variants and higher values corresponding to more somatic mutations. A higher threshold results in a high confidence in the LOH regions, but with missed true regions of LOH, resulting in more founder variants in regions called as non-LOH, while a lower threshold results in over-calling of LOH regions but with less founder variants in the regions called as non-LOH. We tested the calling of LOH regions as described above for all possible thresholds between 0 and 50 SNVs in the 50 heterozygous SNV window. We then divided high confidence somatic mutations into the regions called in these test sets as LOH and non-LOH. We then calculated signature S mutation fraction and dN/dS ratio for each and plotted the values against the threshold used for the test calling (**Extended Data Fig. 2c-e**). Over-calling of LOH drops dramatically before flattening out around 10/50 SNVs, while missed LOH appears to rise consistently as the threshold is increased. Overall, a threshold of 10/50 SNVs

maximizes the difference between somatic mutations in non-LOH vs LOH regions and was used in all other analyses to call LOH regions, so that they could be excluded from somatic mutation analysis.

To validate that our LOH calling method was successfully removing LOH regions we filtered for a different set of SNVs than those used to call LOH: sub-lineage-specific founder variants (variants found in a healthy clam and all individuals of one sub-lineage but none in the other sub-lineage). The density of USA-specific founder variants SNVs was 36× higher in PEI LOH regions versus non-LOH regions, and PEI-specific founder variants SNVs was 20× higher in USA LOH regions versus non-LOH regions (**Extended Data Fig. 2b**), confirming these regions were likely lost from the other sub-lineage.

- 03_SNV_analysis/04a_LOH_calling_upstream.R
- 03_SNV_analysis/04b_LOH_merge_and_helmsman.sh
- 03_SNV_analysis/04c_exclude_LOH_SNVs.sh
- 03_SNV_analysis/04d_LOH_founder_SNV_density.R
- 03_SNV_analysis/06b_run_dNdS.sh
- 03_SNV_analysis/07_LOH_threshold_validation.R

MarBTN phylogeny

- 03_SNV_analysis/01_pairwise_phylogeny.sh
- 03_SNV_analysis/02_initial_SNV_counts.R

Mutational signature extraction and fitting

- 03_SNV_analysis/03a_sig_extraction_upstream.R
- 03_SNV_analysis/03b_sig_extraction_count_trinuc.sh
- 03_SNV_analysis/03c_sig_extraction_fitting_sigfit.R
- 01_Genome_assembly/03a_trinucleotide_counting.sh
- 01_Genome_assembly/03b_trinucleotide_counting.R

Cancer dating

To estimate the total age of the cancer, we first estimated the number of somatic SigS mutations in the trunk of the MarBTN lineage: SNVs shared by all MarBTN samples. We continued to exclude LOH regions and to exclude SNVs shared with any healthy clams. Somatic mutations in non-LOH regions with copy number >1 would have been heterozygous when they occurred, so we filtered for SNVs with an average allele frequency under 0.8 across the 8 MarBTN samples. For comparison, we also analyzed the following SNV bins: likely homozygous SNVs (those with an average allele frequency over 0.8); SNVs in healthy clams; SNVs found in all cancer samples and shared with a healthy sample; and SNVs found in all samples in one sub-lineage but not the other sub-lineage or healthy clams (high confidence somatic mutations). We counted mutations in their trinucleotide contexts and fit the 4 *de novo* extracted signatures as described previously.

The fraction of SNVs found in healthy clams attributable to signature S was taken to be the baseline SigS fraction (0.025). The SigS mutation fraction was near this baseline for individual healthy clam SNVs and for likely founder variants–those found in all MarBTN samples and either shared with a healthy clam or not shared with one of the sequenced healthy clams but homozygous. Heterozygous SNVs found in all MarBTN samples but no healthy samples had noticeably higher SigS fraction (0.056). The difference between this fraction and the baseline was taken to be from SigS mutations in the early somatic evolution of the MarBTN lineage (0.056-0.025=0.031). This fraction is equivalent to 53,350 mutations, or 108 years (95% CI: 48-Inf) by the previous SigS mutation rate calculation. This confidence interval was determined solely from uncertainty in the mutation rate since error estimates from signature fitting with sigfit were negligible in comparison.

Using the SigS mutation estimation for truncal MarBTN SNVs, we also estimated the total number of somatic mutations in each of the MarBTN sub-lineages, which would be a combination of mutations occurring post-MRCA (high confidence somatic mutations) and mutations in the lineage trunk. To first estimate somatic mutations in the trunk, we assumed that somatic SigS mutation fraction had remained steady since oncogenesis at 0.48 (based on high confidence somatic mutations in the two sub-lineages).

Given the following equations describing the total number of mutations being comprised of a fraction of founder SNVs and a fraction of somatic SNVs (a) and that each fraction has a known percentage of mutations due to SigS (b), we then solve for $Fraction_{somatic}$ (c-e):

a) $Fraction_{founder} = 1 – Fraction_{somatic}$

b) $SigS_{observed}$(heterozygous all BTN, no healthy)=$Fraction_{somatic}*SigS_{somatic}+Fraction_{founder}*SigS_{founder}$

c) $SigS_{observed}=Fraction_{somatic}*SigS_{somatic}+ (1 - Fraction_{somatic})*SigS_{founder}$

d) $Fraction_{somatic} = (SigS_{observed} - SigS_{founder}) / (SigS_{somatic} - SigS_{founder})$

e) $Fraction_{somatic} = (0.056-0.025) / (0.48-0.025) = 0.068$

This is equivalent to 116,765 somatic mutations. We then added high confidence somatic SNVs unique to each sub-lineage (those present in all samples from that sub-lineage but none in the other sub-lineage or healthy clams) and corrected for genome size of the non-LOH portion of the clam genome to get mutation density estimates for each sub-lineage (441 and 452 mu/Mb for the PEI and USA sub-lineages, respectively). Note that although we can estimate total mutation count in the lineage trunk, we cannot differentiate individual SNVs as somatic mutations or founder variants.

- 03_SNV_analysis/05a_cancer_dating_prelim.R
- 03_SNV_analysis/05a2_cancer_dating_prelim_withLOH.R
- 03_SNV_analysis/05b_cancer_dating_helmsman.sh
- 03_SNV_analysis/05c_cancer_dating_regression.R
- 03_SNV_analysis/05d_cancer_dating_trunk.R
- 04_CNV_and_SV_analysis/05b_SVs_by_time.R

dN/dS

We calculated global dN/dS, the overall ratio across all genes in the genome, after identifying the following SNV subsets:

- SNVs found in any healthy clam

- SNVs in all MarBTN samples and shared with a healthy clam
- SNVs in all MarBTN samples and not found in any healthy clams in our data set
- SNVs found in all samples for each sub-lineage, but not found in the other sub-lineage or healthy clams. This resulted in three subsets: USA, PEI and SNVs from each sub-lineage combined. These were further filtered to include only SNVs outside called LOH regions.

dNdScv is designed to quantify selection during somatic evolution and corrects for trinucleotide context-dependent biases to estimate a dN/dS ratio normalized to the expected ratio for each gene or the entire genome. dNdScv is designed to be run on datasets of many samples, but in our case we ran it individually on the above SNV subsets. We ran dNdScv with default settings except for setting max_coding_muts_per_sample and max_muts_per_gene_per_sample to 1 billion each, effectively removing these maximum settings, which were designed for conventional cancers. We calculated global dN/dS across the whole genome, including all annotated genes**.** Likely somatic SNVs show largely neutral global dN/dS (0.98, 95%CI: 0.94-1.02), indicating that there is minimal contamination from founder variants, which are assumed to have been predominantly under negative selection, as seen in healthy clams SNVs.

- 03_SNV_analysis/06a_bin_for_dNdS.R
- 03_SNV_analysis/06b_run_dNdS.sh
- 03_SNV_analysis/06c_dNdS_outputs.R

Copy number calling

First we used R package "cn.mops" (v1.32.0) to divide the genome into 1 kB windows and count the number of reads mapping to each window for each of the samples: healthy (3) and MarBTN (8). Any window with low mapping in the reference clam (less than ¼ the average read depth) was excluded from calling as a low-mapping region. Read depth for each window for each non-reference sample was then divided by the reference clam read depth for that window to normalize. Each window was then divided by

the average read depth for that sample to yield a log2 read depth score. We then calculated the median log2 read depth for every 100 1 kB windows to form larger windows of 100 kB.

We then wanted to convert log2 read depth to copy number without prior knowledge of the average ploidy for each sample. We observed distinct peaks corresponding to copy number integers when we plotted histograms of log2 read depth scores genome-wide for each sample. We chose the best fitting average ploidy value for each sample (the value which lined up copy number calls with integer values when multiplied by 2^{log2_score}). This was 3.6 for PEI MarBTN samples, 3.3 for USA MarBTN samples, and 1.9 for non-reference healthy samples. Note that since healthy samples should be diploid, an average ploidy just under 2 is expected, given read mapping will be slightly less efficient for non-reference clams relative to the reference clam (whose reads are mapped to a reference genome built from itself). We multiplied log2 read depth scores by this value to get copy number estimate for each 100 kB window for each sample. Observing close agreement between the samples within each sub-lineage, we calculated the average copy number calls for each sub-lineage. Finally, we smoothed copy number calls in 1 Mb windows to minimize noise in final calls. For each 100 kB window we calculated the standard deviation for the preceding ten 100 kB windows, the following ten 100 kB windows and the surrounding ten 100 kB windows (five 100 kB windows on either side). We replaced the copy number call with the median of the 1 Mb window with the smallest standard deviation, provided the standard deviation was small, defined as less than 1 on the ploidy scale. If the standard deviation was larger than 1 for all windows, we left the original unsmoothed copy number. Finally, we rounded all calls to the closest integer value for the final copy number call for each 100 kB window. However, we kept the unrounded calls for the purpose of visualizing error in our figures (**Fig. 3b** and grey bars in **Fig. 3a**).

To validate our copy number calls, which were based solely on read depth, we used variant allele frequency of somatic mutations. If calls are correct, genome regions that are of a particular copy number should exclusively have certain allele frequencies, such as 0.33/0.67 for CN3 regions or 0.25/0.5/0.75 for CN4 regions. We calculated variant allele frequencies for high confidence somatic mutations, some of which likely occurred after copy number alteration events and therefore should have a frequency

distribution peak around the low frequency value (e.g 0.33 for CN3 or 0.25 for CN4). We separated SNVs specific to each sub-lineage based on the copy number calls at their locations using bedtools (v2.29.1) and calculated average variant allele frequency across each ploidy level in all of the samples. A plot of the variant allele frequency distribution shows that the major peak corresponds to the expected frequency for each copy number bin. There is evidence of some off-target peaks indicating some degree of error in these copy number calls (for example, 0.5 peak in CN3, 0.33 peak in CN2, 0.5 or less in CN1). Some of these peaks indicate regions that are called as lower copy number than the true value (e.g. 0.33 peak in CN2, 0.5 or less in CN1), which is likely due to sequence polymorphism leading to lower mapping than expected. Other off-target peaks, particularly those indicating copy number is called too high, may be due to other causes, such as the confounding effects of repetitive elements in the genome. Overall, copy-number-specific the variant allele frequencies support the conclusion that this copy number calling strategy is accurate and that much of the MarBTN genome has increased in copy number from its diploid founder ancestor.

- 04_CNV_and_SV_analysis/01_CNV_calling.R
- 04_CNV_and_SV_analysis/02_SNVs_by_CNV.sh
- 04_CNV_and_SV_analysis/03_SNV_freq_by_CNV.R
- 04_CNV_and_SV_analysis/03b_CN4_doubling_time.R

Structural variant and telomere calling

- 04_CNV_and_SV_analysis/04_SV_calling_delly.sh
- 04_CNV_and_SV_analysis/05_SV_analysis.R
- 04_CNV_and_SV_analysis/06a_telomeres.sh
- 04_CNV_and_SV_analysis/06b_telomeres.R

Identifying *Steamer* insertion sites

First, we used BWA-MEM to map reads for each sample to the 177 bp *Steamer* long terminal repeat (LTR), a sequence that flanks either side of the internal coding sequence of LTR-retrotransposons. For all reads that mapped, we extracted just the externally flanking portion of each read, discarding reads that

extended into the internal sequence of *Steamer* and discarding the portion of each read that mapped to the *Steamer* LTR. We then mapped these flanking portions to the reference genome, keeping only reads that mapped to a single location in the genome with high confidence (MAPQ score ≥ 30). For reads mapping to the genome with lower confidence (MAPQ score < 30), we rematched each flanking read with its pair and re-mapped to the genome using bwa sampe, and if the mapping of the flanking fragment together with its mate generated a MAPQ score ≥ 30 then it was included as a specific mapped read. Finally, we took all flanking reads that did not map to the genome with high confidence in either step and mapped them to the RepeatModeler2-generated repeat library, since many flanking reads that do not map to a specific site in the genome are likely to be in repetitive regions. We then generated a BED file format for each flanking read mapped to the genome or repeat library, calling each *Steamer* site by its 5 bp target site duplication, which is generated upon insertion and means that upstream and downstream flanking reads will overlap by 5 bp, and whether it was forward or reverse-face relative to the mapped chromosome.

We merged reads by their mapped locations to get the total number of upstream and downstream flanking reads supporting each insertion site, keeping all sites supported by at least five total flanking reads or at least one each of upstream and downstream flanking reads. We corrected for the six *Steamer* insertions that exist in the reference genome, which would otherwise result in upstream and downstream reads mapping 4.7 kB apart if that insertion is present in a sample (before and after the *Steamer* copy in the reference genome), so that upstream and downstream reads were still counted as in support of the same insertion. We estimated total read depth at *Steamer* insertion sites by averaging the read depth 10 bp before and10 bp after the 5 bp target site duplication, only considering reads with MAPQ ≥ 30. We then estimated insertion allele frequency at each site by dividing the number of *Steamer* insertion supporting reads by the total read depth. We then merged these insertion calls for each sample into a single table, converting presence/absence values to 0/1 and creating a distance matrix using dist.gene() and building a neighbor joining tree and bootstrapping using R package "ape" as described for the nuclear phylogeny.

We also counted shared insertion sites between samples (e.g. all MarBTN, PEI only, USA only) as shown on tree branches and reported in the text.

To investigate where *Steamer* inserted relative to genes, we found the closest gene to each insertion site using bedtools closest, excluding insertion sites within genes. There was a noticeable bias in the 1-2 kB upstream genes. To ensure this was not due to read mapping bias, we generated a similar plot based on whole genome sequence read mapping by mapping 0.1% of MELC-2E11 reads to the genome and treating the first 5 bp as a *Steamer* insertion site. This test set did not display this bias. We then counted the number of *Steamer* insertions in annotated regions in the genome (genes, coding sequences, 5'UTR and 3'UTR) in addition to the 1 kB regions upstream of annotated gene regions. We then normalized for both the size of those portions of the genome and how likely reads were to map to these regions (to correct for biases that might skew insertions toward more mappable portions of the genome), yielding the plot found in Figure 4C.

To see whether these genes might be more likely to be cancer-associated, we conducted a blastp search of predicted intact *M. arenaria* gene models for the 729 cancer-associated genes from the COSMIC database, which generated hits of e-value>1e-6 in 14% of *Mya* genes. We then compared the number of *Steamer* insertions that intersect with these genes. In the absence of selection for insertion near these genes we would expect 14% of *Steamer* insertions to intersect with these genes. Observed versus expected insertions were compared with a Chi-squared test.

- 05_TE_analysis/01_identify_steamer_in_ref_genome.sh
- 05_TE_analysis/02_steamer_calling_pipeline.sh
- 05_TE_analysis/03_steamer_downstream_analysis.R
- 05_TE_analysis/04a_steamer_ATG_bias.sh
- 05_TE_analysis/04b_steamer_ATG_bias.R
- 05_TE_analysis/05a_steamer_upstream_bias.sh
- 05_TE_analysis/05b_steamer_upstream_bias.R
- 05_TE_analysis/05c_steamer_upstream_cosmic_bias.sh

TE copy number analysis

- 05_TE_analysis/06a_REPdenovo.sh
- 05_TE_analysis/06b_merge_repeats_and_maps_reads.sh
- 05_TE_analysis/07_TE_coverage_analysis.R

Mitochondrial analysis

We used median allele frequency of cancer-specific mitochondrial SNVs as a proxy for cancer isolate purity and host tissue purity to complement the similar estimation from genomic SNVs. For healthy clams, median allele frequencies were slightly below 100% likely due to sequencing, mapping, or contamination errors, and yield a maximal value for "pure" target DNA. For cancer samples, median allele frequencies were slightly lower, attributed to the presence of host clam DNA, but remained >97%. Two MarBTN isolates that were excluded from this study due to high host DNA contamination were included on this analysis as contaminated sample controls. For samples for which paired tissue sequencing existed (3 of 8 cancer samples), we used the median allele frequency of cancer-specific mitochondrial SNVs to estimate contamination of tissue by cancer DNA. Genomic and mitochondrial SNVs largely agreed in their estimation of percent cancer in hemolymph and tissue, with mitochondria SNVs giving a more precise median estimate due to higher coverage, while nuclear SNVs corrected for potential copy number differences by only focusing on CN2 regions.

We confirmed the presence of a D-loop tandem duplication in a healthy clam using inverse PCR (**Extended Data Fig. 7e)**, with outward facing primers that would only amplify if the copies or the region are in tandem (**Supplementary Table 4**). Amplification of the products of these inverse primers confirms tandem duplication of the region. However, amplicon sizes from primers spanning the D-loop support a single copy of the D-loop. Additionally, the inverse primers spanning the G-rich insertion has a dim band at expected size, but two brighter bands at smaller sizes. Given the highly G-rich region, it is likely that when primers spanning the D-loop are used that the PCR products are recombining to lose the extra copies, with selection in the PCR reaction favoring removal of the G-rich stretch that interferes with amplification.

Given all samples in this study support the presence of tandem D-loop repeats, it is possible that the clam used for the previously published mitochondrial genome that contains a single D-loop copy may have also been multi-copy and missed due to short-read sequences and recombination during cloning to resolve gaps in the mitochondrial genome.

The new reference mitochondrial genome was assembled by taking the previously published mitogenome reference with only a single, collapsed copy of the repeated D-loop region (NC_024738.1), replacing the 696 bp putative repetitive region (12,163-12,857) with a gap of 3×696 Ns, and running PBJelly to fill the gap using PacBio long reads. PBJelly was run to gap-fill the scaffolded assembly using pbsuite (https://github.com/esrice/PBJelly) using using blasr v5.1, networkx 2.2, and Python 2.7 as above, with the protocol file Protocol_MELC.xml. Only captured gaps were filled (no inter-scaffold gaps) using the option "--capturedOnly" during the "support" step. PBJelly was run with the commands:

- Jelly.py setup Protocol_ MELCmtmultifastq.xml
- Jelly.py mapping Protocol_ MELCmtmultifastq.xml
- Jelly.py support Protocol_ MELCmtmultifastq.xml -x "--capturedOnly"
- Jelly.py extraction Protocol_ MELCmtmultifastq.xml
- Jelly.py assembly Protocol_ MELCmtmultifastq.xml -x "--nproc=20"
- Jelly.py output Protocol_ MELCmtmultifastq.xml

Polishing of the mitochondrial genome assembly was done with Arrow (using pbsuite as described above and pbbioconda-0.0.5 with python 3.7). First, the PBJelly output was renamed to PBJelly_mt_genome.fasta, and polishing was run using the commands:

- module load pbsuite/esrice
- pbalign --verbose --nproc 40 /home/metzgerm/MELC-2E11/Marenaria.3.2_bam.fofn PBJelly_mt_genome.fasta MELC-mtalignedall.bam 2>&1 | tee pbalign_stderrout.txt
- module load conda/4.7.10_py3.d/genomicconsensus/2.3.3
- samtools faidx PBJelly_mt_genome.fasta

- arrow --verbose --annotateGFF --reportEffectiveCoverage -j 40 MELC-mtalignedall.bam -r PBJelly_mt_genome.fasta -o MELC-2E11mtvariants.gff -o MELC-2E11mtconsensus.fasta -o MELC-2E11mtconsensus.fastq 2>&1 | tee arrow_stderrout.txt

The polished mitogenome alignment with the completely assembled repeat region (MELC-2E11mtconsensus.fasta) was renamed to mtGenome_PBJelly_polished.fasta.

- 06_Mito_analysis/00_create_coding_dndscv_input.sh
- 06_Mito_analysis/01_mapping_and_SNV_calling.sh
- 06_Mito_analysis/02_host contamination.R
- 06_Mito_analysis/03_dloop_coverage.R
- 06_Mito_analysis/04_SNV_analysis.R
- 06_Mito_analysis/05a_sample_purity_nuclear_and_mito.sh

RNA sequence analysis

- 07_RNA_analysis/01_RNAseq_STAR_alignment.sh
- 07_RNA_analysis/02_RNAseq_analysis.R
