# Supplementary material for: Centuries of genome instability and evolution in soft-shell clam, Mya arenaria, bivalve transmissible neoplasia
Source: Nat Cancer. 2023 Oct 2;4(11):1561–74. doi: 10.1038/s43018-023-00643-7 (PMC10663159; doi:10.1038/s43018-023-00643-7)
Supplement: Supplementary file 2 — Reporting Summary [file 43018_2023_643_MOESM2_ESM.pdf]

Corresponding author(s): Michael J. Metzger

Last updated by author(s): Aug 21, 2023

Reporting Summary

Nature Portfolio wishes to improve the reproducibility of the work that we publish. This form provides structure for consistency and transparency in reporting. For further information on Nature Portfolio policies, see our Editorial Policies and the Editorial Policy Checklist.

Statistics

For all statistical analyses, confirm that the following items are present in the figure legend, table legend, main text, or Methods section.

n/a	Confirmed	
☐	☒	The exact sample size (n) for each experimental group/condition, given as a discrete number and unit of measurement
☐	☒	A statement on whether measurements were taken from distinct samples or whether the same sample was measured repeatedly
☐	☒	The statistical test(s) used AND whether they are one- or two-sided<br>*Only common tests should be described solely by name; describe more complex techniques in the Methods section.*
☒	☐	A description of all covariates tested
☐	☒	A description of any assumptions or corrections, such as tests of normality and adjustment for multiple comparisons
☐	☒	A full description of the statistical parameters including central tendency (e.g. means) or other basic estimates (e.g. regression coefficient) AND variation (e.g. standard deviation) or associated estimates of uncertainty (e.g. confidence intervals)
☐	☒	For null hypothesis testing, the test statistic (e.g. F, t, r) with confidence intervals, effect sizes, degrees of freedom and P value noted<br>*Give P values as exact values whenever suitable.*
☒	☐	For Bayesian analysis, information on the choice of priors and Markov chain Monte Carlo settings
☐	☒	For hierarchical and complex designs, identification of the appropriate level for tests and full reporting of outcomes
☒	☐	Estimates of effect sizes (e.g. Cohen's d, Pearson's r), indicating how they were calculated

Our web collection on statistics for biologists contains articles on many of the points above.

Software and code

Policy information about availability of computer code

Data collection	All programs used are listed in the manuscript methods, and all code is available on GitHub (https://github.com/sfhart33/MarBTNgenome), including all dependencies with version numbers.
Data analysis	All programs used are listed in the manuscript methods, and all code is available on GitHub (https://github.com/sfhart33/MarBTNgenome), including all dependencies with version numbers. Data analysis software included:<br>bedtools (2.29.1)<br>BBTools (38.86)<br>trimmomatic (0.36)<br>bwa (0.7.12)<br>samtools (1.9)<br>somatypus (1.3)<br>helmsman (1.5.2)<br>bcftools (1.10.2)<br>delly (0.8.5)<br>seqtk (1.0)<br>blast+ (2.10.0)<br>CD-HIT (4.8.1)<br>RepeatModeler (2.0)<br>Repeatmasker (4.1.0)<br>REPdenovo (2019.07.20 download)<br>supernova (2.1.1)

FALCON-Unzip (with pbbioconda-0.0.5 and python 3.7)
FALCON-Phase (v0.1.0-beta)
SNAP(version 2006-07-28)
PBSuite (15.8.24, slightly modified: https://github.com/esrice/PBJelly)
blasr (5.1)
networkx (2.2 with Python 2.7)
Longranger62 (2.2.2)
FreeBayes (1.3.1)
Trinity (2.8.5)
MAKER (2.31.10)
exonerate (2.2.0)
BUSCO (v3)
telseq (v0.0.2)
STAR (2.7.5a_2020-06-29)
SAMBLASTER (v.0.1.24)
Juicebox (v1.5.3)

R packages used (R v3.6.0)
Biostrings (2.54.0)
sigfit (2.0.0)
mapdata (2.3.0)
maptools (1.1-1)
tidyverse (1.3.0)
ape (5.5)
lsa (0.73.2)
gridExtra (2.3)
zoo (1.8-8)
geiger (2.0.7)
nlme (3.1-139)
phytools (0.7-90)
dndscv (0.0.1.0)
devtools (2.3.2)
cn.mops (1.32.0)
mixtools (1.2.0)
bedr (1.0.7)
ggseqlogo (0.1)
DESeq2 (1.26.0)
pheatmap (1.0.12)
RColorBrewer (1.1.3)
viridis (0.5.1)
scales (1.2.1)

For manuscripts utilizing custom algorithms or software that are central to the research but not yet described in published literature, software must be made available to editors and reviewers. We strongly encourage code deposition in a community repository (e.g. GitHub). See the Nature Portfolio guidelines for submitting code & software for further information.

Data

Policy information about availability of data

All manuscripts must include a data availability statement. This statement should provide the following information, where applicable:

- Accession codes, unique identifiers, or web links for publicly available datasets
- A description of any restrictions on data availability
- For clinical datasets or third party data, please ensure that the statement adheres to our policy

Raw sequence data and the assembled genome are now fully available via NCBI BioProject PRJNA874712. This study also used the GenBank (KF319019.1, NC_024738.1, GCA_011752425.2, GCF_002022765.2, GCF_002113885.1, GCF_902652985.1, and GCF_902806645.1) and Uniprot (release 2021_01) databases.

Human research participants

Policy information about studies involving human research participants and Sex and Gender in Research.

Reporting on sex and gender	N/A
Population characteristics	N/A
Recruitment	N/A
Ethics oversight	N/A

Note that full information on the approval of the study protocol must also be provided in the manuscript.

Field-specific reporting

Please select the one below that is the best fit for your research. If you are not sure, read the appropriate sections before making your selection.

☒ Life sciences ☐ Behavioural & social sciences ☐ Ecological, evolutionary & environmental sciences

For a reference copy of the document with all sections, see nature.com/documents/nr-reporting-summary-flat.pdf

Life sciences study design

All studies must disclose on these points even when the disclosure is negative.

Sample size	At least three samples of MarBTN were analyzed from each sublineage to enable the comparison of the cancers from each sublineage. No specific sample size calculations were done, but sample size is comparable to other genomic studies of transmissible cancer in the past.
Data exclusions	Sequencing was conducted on BTN samples only from heavily diseased animals, so the cancer samples are highly pure, with minimal host contamination.
Replication	N/A. This study is a genomic analysis of a naturally occurring lineage of transmissible cancer. We have analyzed multiple samples to characterize biological variability, but this is not a laboratory experiment for which replication of the experiment is relevant.
Randomization	Genomic analysis of BTN sequence was conducted through a computational genomic analysis pipeline and all samples were treated equally. As such, randomization is not applicable.
Blinding	Genomic analysis of BTN sequence was conducted through a computational genomic analysis pipeline and all samples were treated equally. As such, blinding is not applicable.

Reporting for specific materials, systems and methods

We require information from authors about some types of materials, experimental systems and methods used in many studies. Here, indicate whether each material, system or method listed is relevant to your study. If you are not sure if a list item applies to your research, read the appropriate section before selecting a response.

Materials & experimental systems

n/a	Involved in the study
☒	☐ Antibodies
☒	☐ Eukaryotic cell lines
☒	☐ Palaeontology and archaeology
☐	☒ Animals and other organisms
☒	☐ Clinical data
☒	☐ Dual use research of concern

Methods

n/a	Involved in the study
☒	☐ ChIP-seq
☒	☐ Flow cytometry
☒	☐ MRI-based neuroimaging

Animals and other research organisms

Policy information about studies involving animals; ARRIVE guidelines recommended for reporting animal research, and Sex and Gender in Research

Laboratory animals	The study did not involve laboratory animals.
Wild animals	Soft-shell clams (Mya arenaria) were collected by hand/shovel at low tide or purchased from commercial sources which collect animals for human consumption (FFM animals from Maine and NYTC animals from New York were purchased, others were field-collected). Adult clams were selected on the basis of size (approximately >1 year), although precise determination of ages is not possible. Animals were transported on ice to a laboratory, where they were diagnosed for the presence of BTN, and samples were taken for sequencing. No animals were returned to the wild.
Reporting on sex	Sex information is not available for all clams and can only be determined through examination of gonads. The reference animal (MELC-2E11) was determined to be female through microscopic analysis.
Field-collected samples	Field-collected samples of animals with BTN that were used in this study (PEI and MELC samples) have been reported previously (Metzger et al. Cell 2015). Healthy animal MELC-2E11 was freshly collected for this study and served as the source of the reference genome. Briefly, all field-collected animals were collected by hand/shovel at low tide, transported to the laboratory on ice, and were housed and maintained in aerated tanks with seawater at 4-16C until diagnosis. The laboratory at PNRI is a terminal quarantine facility, approved by the Washington State Department of Fish and Wildlife (the most recent import permit is # 23-3049). Consistent

with the import permit plan, no animals were returned to the wild. At the end of the experiment, animals were sacrificed, tissue samples were collected (any remaining waste was autoclaved and liquid waste was treated with bleach to disinfect).

Ethics oversight	No ethics approval or oversight is required for invertebrate bivalve mollusks.

Note that full information on the approval of the study protocol must also be provided in the manuscript.
